# Characterizing and Evaluating the Zoonotic Potential of Novel Viruses Discovered in Vampire Bats

**DOI:** 10.3390/v13020252

**Published:** 2021-02-06

**Authors:** Laura M. Bergner, Nardus Mollentze, Richard J. Orton, Carlos Tello, Alice Broos, Roman Biek, Daniel G. Streicker

**Affiliations:** 1 Institute of Biodiversity, Animal Health and Comparative Medicine, College of Medical, Veterinary and Life Sciences, University of Glasgow, Glasgow G12 8QQ, UK; nardus.mollentze@glasgow.ac.uk (N.M.); roman.biek@glasgow.ac.uk (R.B.); daniel.streicker@glasgow.ac.uk (D.G.S.); 2 MRC–University of Glasgow Centre for Virus Research, Glasgow G61 1QH, UK; richard.orton@glasgow.ac.uk (R.J.O.); alice.broos@glasgow.ac.uk (A.B.); 3 Association for the Conservation and Development of Natural Resources, Lima 15037, Peru; carlos.tello.ch@gmail.com; 4 Yunkawasi, Lima 15049, Peru

**Keywords:** Chiroptera, wildlife disease, rabies virus, phylogenetics, machine learning, zoonosis

## Abstract

The contemporary surge in metagenomic sequencing has transformed knowledge of viral diversity in wildlife. However, evaluating which newly discovered viruses pose sufficient risk of infecting humans to merit detailed laboratory characterization and surveillance remains largely speculative. Machine learning algorithms have been developed to address this imbalance by ranking the relative likelihood of human infection based on viral genome sequences, but are not yet routinely applied to viruses at the time of their discovery. Here, we characterized viral genomes detected through metagenomic sequencing of feces and saliva from common vampire bats (*Desmodus rotundus*) and used these data as a case study in evaluating zoonotic potential using molecular sequencing data. Of 58 detected viral families, including 17 which infect mammals, the only known zoonosis detected was rabies virus; however, additional genomes were detected from the families *Hepeviridae*, *Coronaviridae*, *Reoviridae*, *Astroviridae* and *Picornaviridae*, all of which contain human-infecting species. In phylogenetic analyses, novel vampire bat viruses most frequently grouped with other bat viruses that are not currently known to infect humans. In agreement, machine learning models built from only phylogenetic information ranked all novel viruses similarly, yielding little insight into zoonotic potential. In contrast, genome composition-based machine learning models estimated different levels of zoonotic potential, even for closely related viruses, categorizing one out of four detected hepeviruses and two out of three picornaviruses as having high priority for further research. We highlight the value of evaluating zoonotic potential beyond ad hoc consideration of phylogeny and provide surveillance recommendations for novel viruses in a wildlife host which has frequent contact with humans and domestic animals.

## 1. Introduction

Characterizing viruses in wildlife hosts or vectors that have frequent contact with humans and domestic animals is important for understanding viral diversity in nature, as well as identifying novel pathogens that have ecological opportunities to emerge in humans [1]. Metagenomic sequencing has unprecedented power in such investigations, because it can describe viral communities in a relatively unbiased manner, rather than screen for specific taxa [2]. For example, metagenomic surveys of urban rats and mice living in close proximity to humans [3,4] and of human-biting arthropods [5,6] have revealed a variety of viruses that are closely related to human-infecting taxa, which might indicate that they pose heightened risk as zoonoses. While discoveries of novel viruses in wildlife are valuable to understand the host range and distribution of viruses across the tree of life, evaluating the risk of human infection from viral sequence data alone remains largely subjective, often relying on the presence of zoonotic viruses in the same viral family or evolutionary relatedness to known zoonoses or to human viruses. However, such projections may not always be accurate for several reasons. Firstly, closely related viruses can exhibit different pathogenicity. For example, *Cedar henipavirus*, closely related to *Hendra henipavirus*, and *Reston ebolavirus*, closely related to pathogenic ebolaviruses, produce no clinical symptoms in experimental challenge models and in natural infections of humans, respectively, in contrast to their pathogenic relatives [7,8]. Secondly, a remarkably small portion of viral diversity has been characterized and existing taxonomy does not always describe relationships accurately, so sampling biases have the potential to mislead conclusions on zoonotic risk based on phylogeny [9,10,11]. Thirdly, if multiple closely related viruses (i.e., within the same family) are discovered, phylogeny-based recommendations are unlikely to be able to distinguish risk at these finer taxonomic scales. Consequently, if judging the zoonotic risk of newly discovered viruses based on phylogeny alone, resources for follow-up laboratory studies and field surveillance may potentially be misallocated towards taxa which ultimately pose limited zoonotic risk, while legitimate threats may be missed if they are unrelated to already characterized taxa [12]. Additional lines of evidence that help evaluate which viruses discovered at the human–wildlife interface merit further attention could partly address the imbalance between the vast diversity of viruses and the limited capacity for follow-up studies. Models based on the genomic traits of viruses which are not strictly dependent on phylogeny provide one such opportunity. Such models incorporate genomic traits that may contain weak signals, but which can nonetheless be extracted and exploited with machine learning algorithms [13,14,15]. These models, built from exclusively genomic data, have particular potential to contribute to evaluating which viruses merit further study in empirical metagenomic datasets, where the only source of information is genomic data.

Distributed throughout the Americas, common vampire bats (*Desmodus rotundus*) have particularly high contact with other vertebrates due to nightly feeding on mammalian wildlife and livestock [16,17,18], humans [19,20,21], and birds [22]. Local exposures of humans and livestock to vampire bat bites can be high [19,23,24], suggesting that both bat exposure to blood-borne viruses of their prey and prey exposures to viruses shed in bat saliva are probable. Viruses excreted through vampire bat feces could also be transmitted to prey either via direct contact during feeding or through environmental contamination. While vampire bats are best known for their role as a key reservoir of rabies virus in the Americas [21], PCR-based and metagenomic viral discovery efforts have described other vampire bat-associated viruses with unknown zoonotic potential [25,26,27,28,29,30,31]. Vampire bats therefore offer a simplified system for considering zoonotic risk, where viruses not observed to infect humans may reflect either the absence of diagnostics for detection, zoonotic transmission without detectable illness, or the inability of viruses to infect humans, but are unlikely to represent lack of exposure because ecological barriers for zoonotic transmission are minimal. Here, we evaluated the zoonotic potential of novel viruses discovered in vampire bats, employing traditional phylogenetic analyses as well as machine learning models which rank human infection ability based on either the capabilities of similar viruses (i.e., the frequency of zoonotic or human-infecting species within each virus’ “phylogenetic neighborhood” [32]) or on viral genome composition [15]. Our results represent a case study in applying machine learning methods to initially assess the zoonotic potential of viruses at the time of their discovery. Empirical metagenomic datasets commonly recover multiple viruses; therefore, these models offer the opportunity to add additional evidence to evaluations of which viruses, among both closely and distantly related taxa, might be prioritized for future surveillance in the field and in experimental laboratory research.

## 2. Materials and Methods

### 2.1. Datasets

We characterized viruses from metagenomic datasets generated for community-level ecological analyses. Sequenced samples comprised pooled saliva and fecal swabs from vampire bats sampled across Peru (Table S1). We analyzed a total of 62 pools, made up of samples taken from bats in 26 colonies. Of the 62 pools, 16 contained samples from multiple bat colonies, with 16 unique sites in this “multi-colony” dataset [33], and 46 contained samples from one bat colony each, with 23 unique sites in this “single-colony” dataset [34]. Pools from the multi-colony dataset consisted of 10 individual swabs combined across 2 colonies within the same locality (with the exception of one pool, which contained samples from only one colony), while pools from the single colony dataset consisted of up to 10 individual swabs from a colony. In cases where the same virus was detected in both multi and single colony pools from the same locality, which potentially contained samples from the same individuals, only one pool was selected for further analysis.

Bat sampling methods were approved by the Research Ethics Committee of the University of Glasgow School of Medical, Veterinary and Life Sciences (Ref081/15), the University of Georgia Animal Care and Use Committee (A2014 04-016-Y3-A5), and the Peruvian Government (RD-009-2015-SERFOR-DGGSPFFS, RD-264-2015-SERFOR-DGGSPFFS, RD-142-2015-SERFOR-DGGSPFFS, RD-054-2016-SERFOR-DGGSPFFS).

One *Coronaviridae* genome that we assessed here was previously published as a resource for the study of bat-associated coronaviruses [35], but that analysis did not evaluate zoonotic potential. In the present study, we supplemented the RNA dependent RNA polymerase (RdRp) alignment from Bergner et al. [35] with an additional vampire bat *Coronaviridae* sequence from another site and performed machine learning analyses. All other vampire bat viruses analyzed here contributed to community-level metrics of viral diversity [33,34], but have not been previously described or characterized individually below the genus level.

### 2.2. Bioinformatic Analyses

Viral contigs were assembled and classified taxonomically using an in-house bioinformatic pipeline [33]. Briefly, after read trimming and quality filtering [36,37], host reads were removed by mapping to the host genome (Genbank accession PRJNA414273) using bowtie2 [38], ribosomal reads were filtered out using Ribopicker [39], and any remaining reads matching to eukaryotic or prokaryotic RefSeq genomes were identified and removed using Diamond blastx [40]. De novo assembly was then performed on the remaining reads using SPAdes [41], with taxonomy assigned using Diamond for the resulting contigs. Results were visualized with KronaTools [42] and open reading frames were extracted using getORF [43]. For viruses which had incomplete genomes, quality filtered reads from the relevant pool were re-assembled to a closely related full genome representative using bowtie2 to maximize coverage across the genome. Heatmaps and summary data were generated using R, version 3.5.1 [44].

### 2.3. Inclusion Criteria and Study Plan

We focused our analyses on viruses that belonged to viral families containing human-infecting species and had at least one complete genome representative in our dataset (Figure S1; Table S2). This included one known zoonosis, rabies virus (RABV, *Rabies lyssavirus*, family *Rhabdoviridae*), and putatively novel representatives from the families *Hepeviridae*, *Coronaviridae*, *Reoviridae*, *Astroviridae* and *Picornaviridae* with unknown human infectivity. Novel virus genomes were analyzed using phylogenetic and machine learning analyses to evaluate zoonotic potential. These analyses were not carried out for newly generated RABV sequences because we did not recover a full genome from metagenomic data. Instead, as a positive control for the machine learning analyses, we evaluated a published genome of vampire bat RABV (Genbank accession EU293133) which was most similar to lineages circulating in Peru as determined by a nucleotide blast against Genbank. We also performed several additional analyses evaluating the prevalence of active RABV infection in vampire bats using RT-PCR and Sanger sequencing (Results Section 3.1).

### 2.4. Phylogenetic Analysis

Nucleotide and amino acid sequences were aligned with published reference sequences (Table S3) using MAFFT [45] within Geneious v.7.1.7 [46]. The best substitution model was selected using jModelTest [47] for nucleotide alignments and ProtTest3 [48] for amino acid alignments. Phylogenetic analyses were performed using maximum likelihood inference in RAxML using the rapid bootstrapping algorithm [49]. Phylogenetic trees were visualized in R using the packages ape, phytools, phangorn, and ggtree [50,51,52,53].

### 2.5. Machine Learning Analyses

Viruses were ranked by their predicted probability of being able to infect humans using two machine learning models, described in Mollentze et al. [15]. Firstly, zoonotic potential was evaluated using a model that solely considers the phylogenetic neighborhood (PN) of the novel virus. This model is a quantitative implementation of the common assumption that viruses which are closely related to those that infect humans are more likely to be zoonotic. For the PN model, each novel virus was analyzed by nucleotide blast against all viruses in the model’s training dataset. The machine learning model then predicted the probability of infecting humans based on a summary of the proportion of matches to known human-infecting viruses and the genetic distance to such matches. Secondly, we evaluated zoonotic potential using the best performing model from Mollentze et al. [15], referred to here as genome composition-based (GCB), which relies on a range of summary statistics describing the viral genome features and their similarity to the same characteristics of human genes (human similarity features). Viral genome composition was characterized by calculating amino acid frequencies, the level of preference for certain codons over alternatives encoding the same amino acid (codon usage bias), and the level of over-representation of specific dinucleotide pairs at various points in the genome (dinucleotide bias, calculated genome-wide, in open reading frames, at bridges between adjacent codons, and at non-bridge positions). Compositional similarity to specific hosts is the most commonly hypothesized explanation for the distinctive genome compositional patterns observed across unrelated viruses [54,55,56,57]. We calculated human similarity features relative to human interferon stimulated genes (ISGs, likely to be expressed during viral infection), non-ISG housekeeping genes (expressed in all cells and tissue types), and remaining genes. For both model types, predictions were obtained from the best 100 out of 1000 models trained using data from 1000 randomly sampled subsets of 732 virus species with known human infectivity (assuming viruses not reported from humans were non-zoonotic). None of the novel viruses described here were included in model training or validation. Following Mollentze et al. [15], the predicted probabilities from these models were used to categorize viruses into four possible priority categories with reference to the proportion of viruses (0.303, here referred to as the cutoff value) which are known to infect humans across 36 animal-infecting virus families which were included in the initial model development. Using this cutoff value represents the best balance between sensitivity and specificity for these models [15]. In this scheme, viruses predicted to be more likely to infect humans than the average known virus are classified as either very high priority (entire 95% confidence interval (CI) above cutoff value), high priority (mean prediction > cutoff, but CI includes values below the cutoff), or medium priority (mean prediction ≤ cutoff, but CI includes values above the cutoff). Viruses for which ≥95% of models predicted a probability of being able to infect humans below that of the average virus (i.e., entire 95% CI < cutoff value) were classified as low priority for future research. To investigate the basis for the predictions of the GCB model, we also calculated the relative contribution of each genome composition measure to the predicted probability that each virus may infect humans (i.e., the effect size) as described in Mollentze et al. [15] and Lundberg et al. [58].

## 3. Results and Discussion

**Figure 1:**
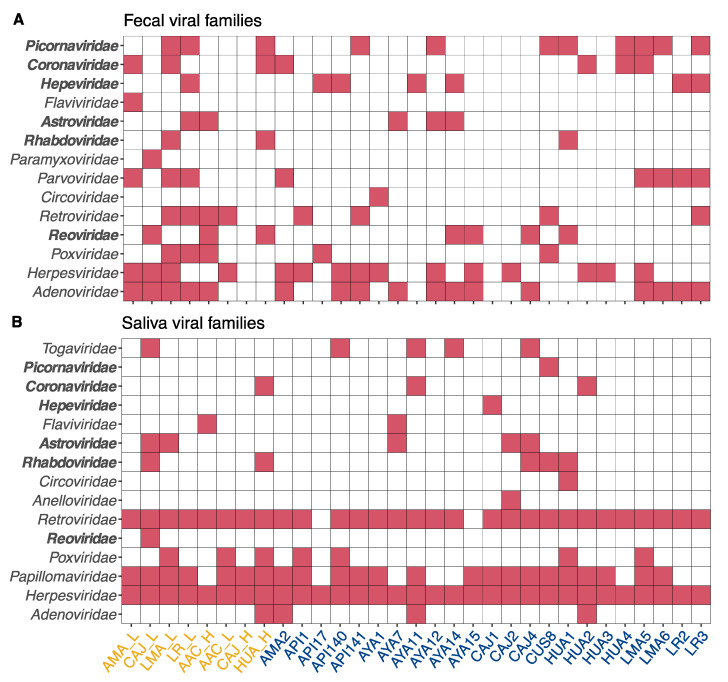
Heatmaps showing the presence (red) or absence (white) of contigs matching to mammal-infecting viral families found in (**A**) feces and (**B**) saliva pools from vampire bat metagenomic datasets. Pools are named and colored according to original studies in yellow [33] and blue [34]. Viral families studied in depth are indicated in bold.

The combined multi-colony and single colony datasets contained contigs from 58 viral families (136 genera), of which 17 families (34 genera) were known to infect mammals (Figure 1). Other viral families represented bacteriophages and viruses that primarily infect plants, arthropods, or other non-mammalian taxa. Viral families detected in feces at a given site were not always detectable in saliva, and vice versa (Figure 1). This discrepancy likely reflects differences in the tissue tropism of different viral groups infecting vampire bats.

### 3.1. Rabies Virus

Vampire bats are an important regional reservoir for rabies virus, a negative sense, single stranded RNA virus. Vampire bat RABV is endemic to regions of Peru east of the Andes and in the Amazon [59]. Accordingly, six metagenomic pools contained contigs matching to the genus *Lyssavirus*, which were confirmed as RABV by nucleotide blast against Genbank [60]; two additional pools contained contigs from the family *Rhabdoviridae* which were identified as genus *Vesiculovirus*. The six pools containing RABV contigs were unlikely to represent independent detections, because they originated from three multi-colony and three single colony pools containing extracts from some of the same individuals. We therefore focused only on the single-colony dataset, in which RABV was detected in two out of 23 colonies (colonies CAJ4 and HUA1), including two saliva pools (CAJ4_SV and HUA1_SV) and one fecal pool (HUA1_F). To evaluate rabies prevalence, we screened the ten individuals that had been included in each single colony pool positive for RABV using an RT-PCR targeting the nucleoprotein gene [61]. Positive samples were Sanger sequenced (Eurofins Genomics) and aligned with previously published RABV sequences from Peruvian livestock [59].

RT-PCR analysis of samples from all individuals included in these metagenomic pools revealed that two individuals, one from each colony, were RABV-positive. The same individual (bat #8012) from colony HUA1 had identical rabies virus RNA sequences in saliva and fecal swabs (100% identity over 688 bp). Comparison of the vampire bat sequences with sequence data from livestock revealed that they belonged to a common Peruvian vampire bat RABV lineage which spills over into domestic animals (nucleotide identity to lineage L1 sequences from Streicker et al. [59]: HUA1_SV 96.0–99.9%, CAJ1_SV 96.4–99.5%).

Although we did not recover a full RABV genome from metagenomic sequencing and were therefore unable to perform machine learning analyses on newly generated sequences, a published vampire bat-associated RABV genome was ranked as medium priority by the PN model (mean calibrated score 0.3 (0.25–0.38)) and very high priority by the GCB model (0.7 (0.41–0.94)), in agreement with past predictions for this virus species [15].

The two localities positive for RABV have historical evidence of rabies circulation, including previous serological evidence of rabies at colony CAJ4 [24,62]. Although individual rabies detections in saliva were globally low (two out of 306 individuals included in metagenomic pools), presence of RNA in one out of ten individuals at two colonies is consistent with a model in which the virus is maintained at low prevalence at the metapopulation level, but at the local level undergoes periods of much higher prevalence due to extinction/recolonization dynamics [63,64].

Detecting RABV in saliva samples from wild vampire bats using metagenomics is methodologically novel, demonstrating a new method of detecting rabies infections, although perhaps unsurprising given that RABV is known to be transmitted via saliva of infected animals. Although *Lyssavirus* RNA has been reported in the feces and organs of bats [65,66], ours is the first evidence of rabies virus in fecal samples from vampire bats. Our results suggest that using non-invasive sampling and molecular surveillance for rabies, which could include metagenomics or less expensive methods such as PCR, may be more practical than previously believed, particularly given that local prevalence can apparently periodically rise to high levels.

### 3.2. Hepeviridae

**Figure 2:**
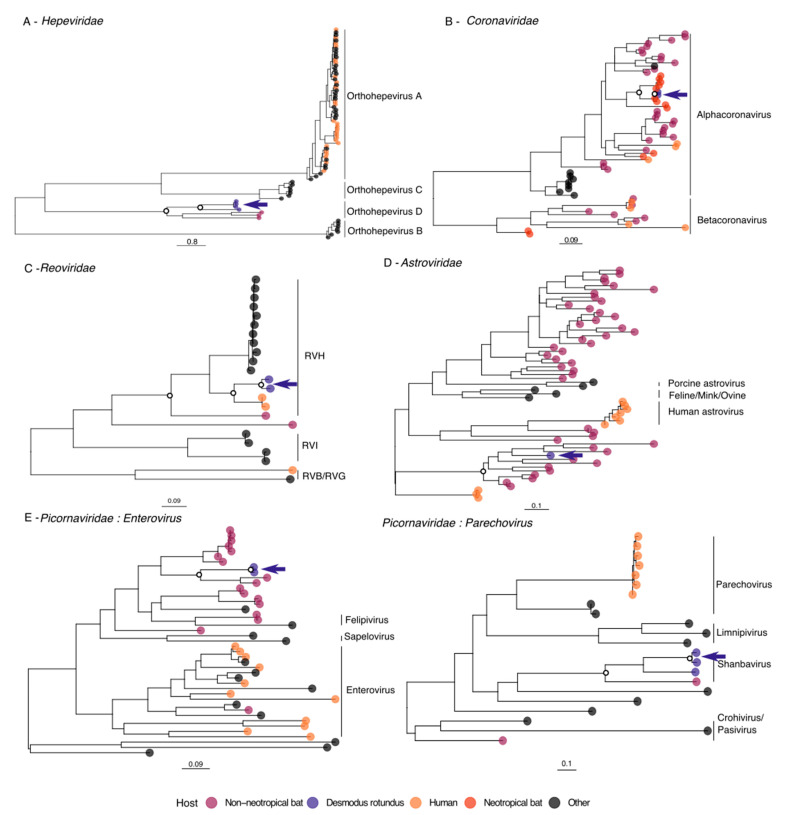
Phylogenetic placement of novel vampire bat viruses. Maximum likelihood phylogenies show relationships of vampire bat viruses (indigo) with previously described viruses from five different families. Two separate phylogenetic analyses were performed for *Enterovirus*-like and *Parechovirus*-like viruses in the family *Picornaviridae*. International Committee on Taxonomy of Viruses (ICTV) recognized viral species or genera are shown on the right side of trees; some *Rotavirus* taxa are not yet officially recognized, but are shown to provide context for new sequences. Bootstrap support values >90 are shown as white circles for key nodes. Arrows indicate vampire bat viruses. The scale bar represents the mean number of substitutions per site. Detailed versions of each phylogeny including node support and Genbank accessions for published sequences are shown in Figures S2–S7.

Hepatitis E viruses (HEV; family *Hepeviridae*) are positive sense, single stranded RNA viruses which are enterically transmitted and cause the most common form of acute hepatitis in the world [67,68]. HEV-like contigs were detected in fecal samples from six vampire bat colonies, four of which yielded near complete sequences and were selected for phylogenetic analysis (AYA11_F, 6645 bp; AYA14_F, 6647 bp; API17_F, 6632 bp; LR3_F, 6657 bp). A full genome nucleotide phylogeny showed that vampire bat HEV sequences formed a monophyletic group (bootstrap support (BS) = 100%) within the *Orthohepevirus D* group along with other bat viruses (Figure 2A and Figure S2). An RdRp amino acid phylogeny, which allowed inclusion of one sequence from a Neotropical bat, confirmed that bat sequences comprised a monophyletic clade distinct from other mammalian HEVs, although placement of the bat clade differed relative to the full genome tree (Figure S3). The placement of the bat clade in the full genome tree agreed with Drexler et al. [69], in which all mammalian HEVs including bat HEVs share a common ancestor, while the RdRp tree suggested bat HEVs are most closely related to avian HEVs [70]. Regardless of placement, novel vampire bat HEVs form part of the apparently bat-specific *Orthohepevirus D* species [69]. Therefore, a manual evaluation based on phylogeny alone would likely characterize these viruses as low priority.

**Figure 3:**
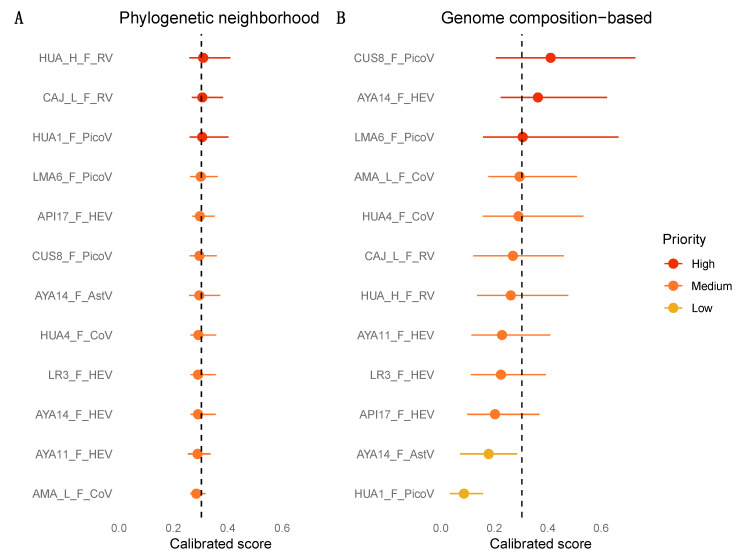
Prioritization of novel viruses based on zoonotic risk score. The figure shows the predicted probability of human infection for each novel virus based on (**A**) the phylogenetic neighborhood model and (**B**) genome composition-based model. Viruses are colored and ordered according to predicted probability of infecting humans. Points show the mean calibrated score, with lines indicating 95% confidence intervals. The dashed line indicates a cutoff of 0.303, which balances sensitivity and specificity. Predicted scores and confidence intervals from machine learning models were used to categorize viruses into four possible priority categories.

The PN model indicated the four novel HEVs as medium priority for further research, but all predicted probabilities were similar and close to the cutoff value (Figure 3A). The GCB model classed one virus, AYA14_F, as high priority while the other three HEVs were medium priority (Figure 3B); these predictions are broadly in agreement with past predictions for *Orthohepevirus D* (medium priority [15]). Disagreement between predictions for different genomes, combined with the fact that only a small fraction of existing diversity has thus far been characterized (including only five recognized HEV species, one of which widely infects humans), means we cannot exclude the possibility of zoonotic transmission for vampire bat HEVs.

HEV variants are geographically widespread in humans, as well as in wildlife, with the species *Orthohepevirus A* representing an ongoing zoonotic threat in many regions [70,71]. Bat HEVs appear to have a stable association with their hosts, primarily grouping monophyletically in the *Orthohepevirus D* species [69,72,73], with vampire bat HEVs being new representatives of this species. However, the rodent-associated HEV species *Orthohepevirus C*, previously thought exclusive to rodents, has been detected in humans [74], emphasizing that divergent HEV species should not be discounted as potential zoonoses.

### 3.3. Coronaviridae

Coronaviruses (CoVs; family *Coronaviridae*) are positive sense, single stranded RNA viruses which occur in diverse host species but are particularly common in bats, where they exhibit high levels of genetic diversity [75,76]. A CoV-like contig of full genome length (29,097 bp) was detected in one multi-colony fecal pool from the department of Amazonas (AMA_L_F) and has been described previously [35]. Several contigs adding up to a near-complete genome (29,065 bp) were also detected in one single-colony fecal pool from a different region of Peru (HUA4_F). Phylogenetic analysis of the RdRp revealed that vampire bat sequences from Peru grouped together monophyletically (BS = 90%) and fell within a well-supported clade (BS = 83%) of *Alphacoronavirus* sequences from other Neotropical bats (Figure 2B and Figure S4). The vampire bat CoVs grouped within a clade of as yet unclassified *Alphacoronaviruses* associated with bats in the family *Phyllostomidae*, highlighting a rapidly expanding knowledge of coronavirus diversity in this group. Because none of the viruses in the Neotropical bat CoV clade are currently known to infect humans, an approach based on the current phylogeny would be expected to assign relatively low priority to vampire bat CoVs.

Both the PN and GCB models classed the two vampire bat CoVs as medium priority for further study, although the relative priority of CoVs compared to other vampire bat viruses was higher for the GCB model (fourth and sixth priority) compared to the PN model (Figure 3).

The zoonotic potential of bat CoVs is noteworthy, with several human-infecting taxa having putative bat origins [77,78,79]. Diverse CoVs exist in Neotropical bats [80,81,82], and there have been several reports of CoVs in vampire bats [25,35,83]. While the most noteworthy CoVs are in the genus *Betacoronavirus*, pathogenic human (HCoV-NL63, HCoV-229E) and animal (SADS-CoV, PEAV) *Alphacoronaviruses* have suspected bat origins [84,85,86]. We only detected full genomes of CoVs in vampire bat feces, but CoV contigs were also present in saliva (Figure 1), suggesting the possibility of oral transmission, although it is unknown whether these detections represent infectious material. Transmission to humans via feces is also conceivable, either via direct exposure to contaminated environments or via intermediate hosts, which are suspected to have played a role in the emergence of some zoonotic bat-derived CoVs (SARS-CoV, MERS-CoV). Given the relatively high ranking of vampire bat CoVs compared to other vampire bat viruses, past emergence of *Alphacoronaviruses* from bats, and high contact between vampire bats and other hosts, we suggest these viruses should not be discounted from further study.

### 3.4. Reoviridae

Rotaviruses (RVs, family *Reoviridae*) are segmented double stranded RNA viruses which cause acute diarrhea in humans as well as other mammals and birds. The common and geographically widespread antigenic group Rotavirus A (RVA )has a wide host range including bats [87,88], while studies have also described uncommon Rotavirus H (RVH) and Rotavirus J (RVJ) antigenic species in bats [89,90,91]. RV-like contigs were detected in four single-colony fecal pools and three multi-colony fecal pools, two of which (CAJ_L_F, HUA_H_F; segment lengths in Table S4) contained contigs matching to ten (of typically eleven) genome segments. Percent identities between the two vampire bat viruses were variable across segments, and both viruses appeared to be missing the VP7 segment, suggesting that it may be either absent or undetectable by metagenomic sequencing (Table S4).

Phylogenetic analysis of the VP6 protein sequence [92] revealed that the vampire bat sequences were most similar to the antigenic group RVH (Figure 2C and Figure S5), a relatively poorly known group which includes strains isolated from humans and pigs [93,94,95,96], as well as bats from South Korea and Cameroon [89,91]. Vampire bat-associated RVH sequences formed a monophyletic clade (BS = 100%) within another well-supported clade containing two human-associated RVH species (BS = 97%). The most closely related bat-associated RVH from South Korea had only small sequence fragments available for segments VP1, VP2 and VP4; comparison of pairwise similarities showed relatively low similarity (58–66.2% nucleotide identity) with vampire bat viruses (Table S4). The RVH phylogeny suggested a lack of monophyly among bat viruses and the possibility of historical transmission between species, both of which have been noted in previous studies of bat RVAs [87,97,98]. The close relationship between vampire bat RVH and two human RVH sequences in the phylogeny suggests that, based on this approach, the vampire bat RVH viruses should be considered likely candidates for zoonotic transmission.

The PN model predictions indicated both vampire bat RVs as high priority, in agreement with what would be concluded based on the phylogeny. In contrast, the GCB model indicated both genomes as medium priority (Figure 3). Given that the RVH group remains relatively poorly studied, there is a strong likelihood of unsampled viruses, such that the apparently close phylogenetic relationship between bat and human RVH sequences is only tentative. Indeed, our detection of RVH in vampire bats represents the first report of this antigenic type in any wildlife species in the Americas. Observations of zoonotic transmission and reassortment between human and animal viruses in the well-studied RVA group suggest that these phenomena might also occur in poorly known RV groups [88,97,98,99]. However, the GCB classification of the RVH genomes as medium priority, in agreement with RVH sequences analyzed in a previous study [15], suggests that evaluations based on the current phylogenetic placement closest to human-infecting viruses are likely to overestimate zoonotic potential.

### 3.5. Astroviridae

Astroviruses (AstVs, family *Astroviridae*) are positive sense single stranded RNA viruses which cause gastroenteritis in humans, as well as in diverse mammals and birds [100]. AstVs occur in bats with high prevalence and genetic diversity, and these infections appear minimally pathogenic to their hosts [76,101]. A full-genome length AstV-like contig (5310 bp) was found in pool AYA14_F, while smaller contigs were detected in several other pools.

Phylogenetic analysis was performed on the RdRp from the AYA14_F genome along with other mammalian AstV sequences, revealing that the vampire bat AstV fell within the genus *Mamastrovirus* in a well-supported clade of bat viruses (BS = 91%) from Asia and Europe (Figure 2D and Figure S6). The vampire bat AstV grouping with diverse bat hosts from Europe and Asia suggests an ancient relationship between bat AstVs from different parts of the world [101,102]. Although presence of AstV in Neotropical bats has been reported [31], there were no published Neotropical bat-associated AstV sequences, so we could not evaluate the relationship of the vampire bat AstV to other Neotropical bat viruses. The PN model indicated the AstV as medium priority for further research, while the GCB model classed the virus as low priority (Figure 3).

Mammalian AstV were long thought to be host-specific, but this finding has been challenged by evidence that non-human primates can be infected with human AstV strains [103], apparent transmission of a canine AstV to humans [104], and the discovery of a mammalian-like AstV in an avian host [105]. Although there are no known instances of bat-to-human AstV transmission, this may be due to lack of surveillance [106], highlighting the importance of evaluating zoonotic potential for viruses in hosts with high interspecific contact. In agreement with previous studies inferring low zoonotic potential for AstVs [107,108], the phylogenetic placement of the vampire bat AstV, along with both PN and GCB models indicated that the vampire bat AstV was medium to low priority for further research, providing an example of a virus in which both current knowledge of this family and all three methods of zoonotic risk assessment led to the same conclusion.

### 3.6. Picornaviridae

Picornaviruses (PicoVs, family *Picornaviridae*) are positive sense, single stranded RNA viruses that infect a wide range of host species, including bats [109,110,111,112,113]. Two groups of PicoV-like viruses were found in fecal pools; contigs from pools HUA1_F (3313 bp) and HUA4_F (758 bp) were similar to an unclassified group of bat PicoVs related to *Enterovirus* [109,113] while contigs from pools API141_F (429 bp), AYA12_F (429 bp), LMA5_F (833 bp), LMA6_F (6868 bp) and CUS8_F (6774 bp) were similar to the genus *Parechovirus*.

Separate phylogenetic analyses were performed for *Enterovirus*-like sequences and *Parechovirus*-like sequences. For *Enterovirus*-like sequences, phylogenetic analysis was performed on a fragment of the 3D polymerase genome region encoding the RdRp, which had been characterized in bat species [113]. The HUA1_F and HUA4_F sequences formed a monophyletic group (BS = 100%) and grouped within a well-supported clade (BS = 99%) of bat-associated PicoVs from across Europe and Asia (Figure 2E and Figure S7A). Bat viruses are not as well-known from the *Parechovirus*-like part of the PicoV family and no part of the genome has been used in any amplicon-based study in bats; the most similar sequence was also generated from a metagenomic study [112]. Therefore, for the phylogenetic analysis, all closely related genomes from the ICTV *Picornaviridae* family tree were included [114], as well as closely matching nucleotide blast hits. The *Parechovirus*-like contigs grouped monophyletically with one another (BS = 100%) and formed a well-supported clade with a virus classified as *Shanbavirus A* (BS = 100%; Figure 2E and Figure S7B), a bat-associated PicoV from China [112]. The *Parechovirus*-like contigs are therefore likely new representatives of the genus *Shanbavirus*. The PicoV contigs from API141_F and AYA12_F did not contain sufficient overlapping sequence to be included in the phylogeny, but comparing a region of the genome shared by all vampire bat viruses showed that API141_F and AYA12_F were most closely related to one another, followed by the LMA viruses (Table S5).

The PN model predictions indicated the HUA1_F *Enterovirus*-like virus as high priority, while the GCB model indicated it was the lowest priority vampire bat virus (Figure 3). Both of the two near-complete genomes of *Parechovirus*-like viruses detected (LMA6_F and CUS8F) were ranked as medium priority by the PN model. In contrast, the GCB model classified both *Parechovirus*-like viruses as high priority, with CUS8F ranking as the highest priority among all novel viruses analyzed here.

PicoVs have not been reported in Neotropical bats before, although there would be no reason to suspect their absence given a wide distribution across Old World and North American bats [110,111,112,113]. Zoonotic PicoV taxa have been previously described; for example, rodents are thought to serve as reservoir hosts for human-infecting PicoVs [3,115,116]. While there are no known instances of bat-to-human PicoV transmission, and rodent PicoVs have been found more likely to be zoonotic relative to PicoVs from other reservoirs [108], it is plausible that bat PicoVs also have zoonotic potential. Our analysis highlights the value of considering distinct viruses from within the same family separately; HUA1_F was ranked the lowest of all novel viruses, while CUS8_F was ranked highest. Despite being in a relatively poorly characterized genus of the PicoV family, both *Parechovirus*-like viruses assessed were ranked among the top three highest priority of all the novel viruses detected in our study based on their genome compositional features, illustrating that having little information about closely related viruses should not preclude novel viruses from being considered a research priority.

### 3.7. Zoonotic Ranking

**Figure 4:**
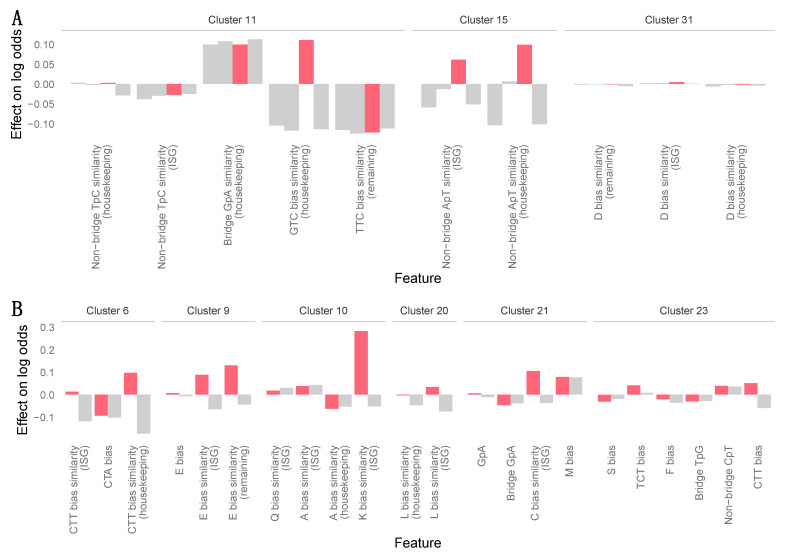
Compositional features influencing predictions of the GCB model. Features are grouped in discrete clusters of correlated features. (**A**) Effects of individual features making up key clusters which explain the difference in priority between AYA14_F_HEV (red) and other HEVs (grey). (**B**) Individual feature effects for key clusters explaining the differences in scores assigned to CUS8_F_PicoV (red) and LMA6_F_PicoV (grey), the two *Parechovirus*-like *Picornaviridae* for which near complete genomes were available. Feature clusters were taken from Mollentze et al. [15], and the overall influence of each cluster on predictions for a given virus was calculated by summing effect sizes across all features in the cluster (see Figure S8). Each illustrated cluster increased the predicted log odds for the virus highlighted in red (the highest-ranked virus in each group) and had an effect on this virus which was more than five-fold larger than its effect on any other closely related virus. Features containing the word “similarity” measure compositional similarity between each virus and a set of human genes (interferon stimulated genes (ISG), non-ISG housekeeping genes, or remaining genes). All other features describe virus genome composition directly. Names starting with a single letter (e.g., “D bias”) describe amino acid usage biases, names with three capital letters (e.g., “GTC bias”) describe codon usage biases, and the remaining features (e.g., “TpC” bias) describe over- or under-representation of specific dinucleotides, measured either across the whole genome or specifically at codon bridge or non-bridge positions.

Prioritization of novel vampire bat viruses based on zoonotic potential differed between the two machine learning models. The PN model yielded little predictive information, generating probabilities that were very close to the cutoff (0.303) which represented the baseline probability expected from viruses in the 36 animal-infecting viral families included during model training (Figure 3A). In contrast, the GCB model classed three novel vampire bat viruses (CUS8_F_PicoV, LMA_6_PicoV and AYA14_F_HEV) as high priority for future research, while other novel viruses were assigned as medium or low priority (Figure 3B). As expected, GCB predictions differed from what might have been concluded based on the qualitative evaluation of phylogenies or the PN prediction. For example, the only viral family in which novel vampire bat viruses grouped most closely with a human clade was *Reoviridae*, and the PN model accordingly ranked these viruses as high priority. In contrast, the GCB model classed these viruses as only medium priority. Intriguingly, one high priority virus grouped phylogenetically with other vampire bat viruses that were classed as medium priority. The HEVs AYA14_F, AYA11_F and API17_F formed a well-supported clade and were 88.4–94.9% similar at the nucleotide level across the genome. However, AYA14_F_HEV was deemed high priority, while the other two HEVs ranked considerably lower amongst all viruses detected here and were classified as medium priority (Figure 3). These differing predictions were driven by differences in a few key measures of genome composition, which resulted in differences in the amount of compositional similarity which these viruses showed to human genes (Figure S8). The largest of these differences related to ApT dinucleotide usage and GTC codon usage bias (Figure 4A). Compositional differences between the two *Parechovirus*-like PicoVs were more widespread (Figure 4B and Figure S8) but resulted in only a moderate difference in predicted scores (Figure 3).

In Mollentze et al. [15], the GCB model performed considerably better than the PN model (AUC = 0.77 and 0.61, respectively). AUC scores within viral families were variable, and sometimes unreliable due to small samples sizes, but gave an indication of how well the model was able to rank human infecting viruses above other viruses from the same family. The family-level analyses indicated that the model performed less well for *Reoviridae*, with an AUC score of 0.47 (0.28–0.66), and could not be calculated for *Hepeviridae* given the small number of known species in this family, suggesting that predictions for these two families should be interpreted with caution [15]. However, the model is known to perform well for all other viral families studied here, with AUC scores >0.7 for *Coronaviridae*, *Astroviridae*, and *Picornaviridae*. More generally, all our evaluations of zoonotic potential, including both phylogenetics and machine learning, were performed with incomplete knowledge of viral diversity, and predictions should be re-evaluated as gaps are filled. Our results emphasize the importance of continued viral discovery, as well as downstream experiments evaluating the capacity of novel animal viruses to infect human cells, which could in turn improve the predictive power of models.

## 4. Conclusions

Given the rapid rate of discovery of novel wildlife viruses, many of which lack reliable ecological and phenotypic data, it is important to focus limited downstream research and surveillance resources on those most likely to pose a threat of emergence in humans. Although the quantitative risk assessments required for this are still in their infancy, we demonstrate a case study using both phylogenetics and machine learning models to initially assess viruses detected in vampire bats, a wildlife species in close and frequent contact with humans and domestic animals. The viruses characterized in our study represent a snapshot of the current viral community in vampire bats, and the GCB model allows us to prioritize those viruses accordingly. Periodic snapshots of viral diversity would allow us to monitor viral communities over time, potentially prioritizing differently in the future. In the meantime, we can focus research efforts on the highest priority viruses, which are closest to being able to infect humans now.

Rabies virus was the only known zoonosis detected, and although it is well-known from vampire bats, our sporadic metagenomic detection of the virus in saliva and feces of wild bats suggests the possibility of molecular surveillance in the field and reinforces the emerging consensus that RABV undergoes outbreak and extinction dynamics within bat colonies [63,64]. GCB analyses of novel vampire bat viruses in the families *Hepeviridae, Coronaviridae*, *Reoviridae*, *Astroviridae* and *Picornaviridae* indicated most of these novel viruses are medium to low priority for further research. This allows us to focus our attention on a considerably reduced set of viruses: only one HEV and two PicoVs were classed as high priority. Our analysis of an empirical dataset also revealed variability in prioritization among closely related viruses, highlighting that viruses should not be excluded as research priorities because one representative appears to pose low risk.

Given the current lack of virus characterization from most mammalian host species and the resulting sparsity in most viral phylogenies (Figure 2), insights from the GCB model would not have been possible based only on information from phylogenies or the PN model, but this information can now be used to prioritize these viruses in follow-up studies. Future studies could include functional characterization in the laboratory, particularly evaluating the capacity of novel viruses to infect human cells, which could be used to validate predictions made by the GCB model. Another important avenue for future work is studying virus epidemiology and evolution in bat populations, which could potentially include evaluating rates of viral evolution and recombination in wild bats and monitoring animals ecologically connected to vampire bats which might serve as intermediate hosts. Although one HEV and two PicoVs ranked above all other novel taxa discovered in terms of their importance for future research and monitoring, we also recommend maintaining research and surveillance efforts on rabies virus, a recognized economic and public health concern for which vampire bats serve as a key reservoir.

## Data Availability

Metagenomic sequence datasets analyzed here are available at the European Nucleotide Archive under Projects PRJEB28138 (https://www.ebi.ac.uk/ena/browser/view/PRJEB28138) and PRJEB34487 (https://www.ebi.ac.uk/ena/browser/view/PRJEB34487). Viral genome and partial genome sequence are available on Genbank (Accessions MT663548; MW249010-MW249040; MW259060- MW259064; see Table S2).

## Supplementary Materials

The following are available online at https://www.mdpi.com/1999-4915/13/2/252/s1. Figure S1: Schematic depiction of novel viral genomes discovered in vampire bats; Figure S2: *Hepeviridae* full genome phylogeny; Figure S3: *Hepeviridae* RdRp phylogeny; Figure S4: *Coronaviridae* RdRp phylogeny; Figure S5: *Reoviridae* VP6 phylogeny; Figure S6: *Astroviridae* phylogeny; Figure S7: *Picornaviridae* phylogenies; Figure S8: Effect of discrete clusters of correlated features on predicted scores for *Hepeviridae* and *Parechovirus*-like *Picornaviridae*; Table S1: Metagenomic datasets from which novel vampire bat viruses were characterized; Table S2: Viral sequences examined in this study, sampling details, and associated Genbank and ENA accessions; Table S3: Genbank accessions, viral family and host for viral taxa included in phylogenies; Table S4: Pairwise identities for each segment between novel vampire bat RVH sequences, the closely related human RVH B219, and bat RVH which was not included in the phylogeny; Table S5: Similarities between *Parechovirus*-like sequences in vampire bats from different locations.
