# Supplementary material for: Characterizing and Evaluating the Zoonotic Potential of Novel Viruses Discovered in Vampire Bats
Source: Viruses. 2021 Feb 6;13(2):252. doi: 10.3390/v13020252 (PMC7914986; doi:10.3390/v13020252)
Supplement: Supplementary file 1 [file viruses-13-00252-s001.pdf]

Supplementary Figures

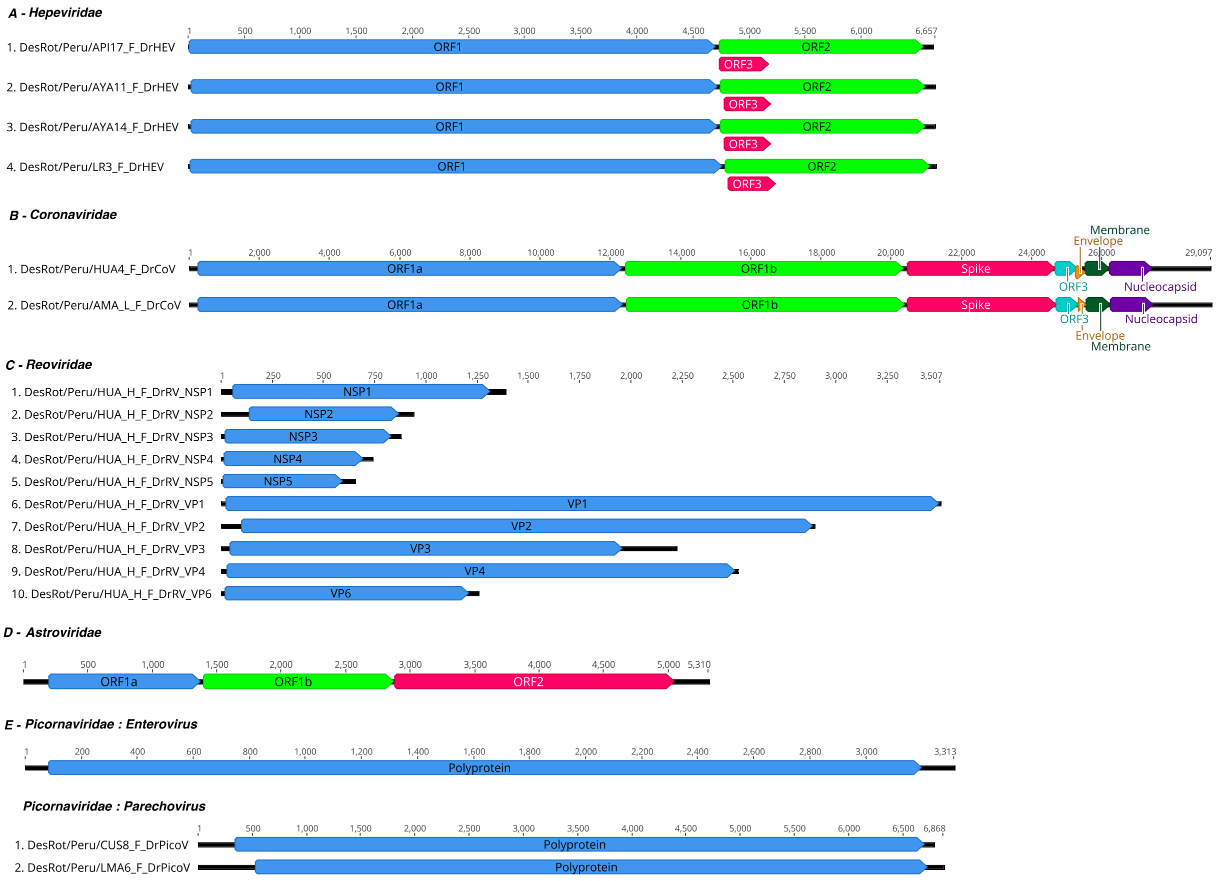

Figure S1. Schematic depiction of novel viral genomes discovered in vampire bats. Open reading frames are represented as colored arrows. Only complete or near complete genomes are shown.

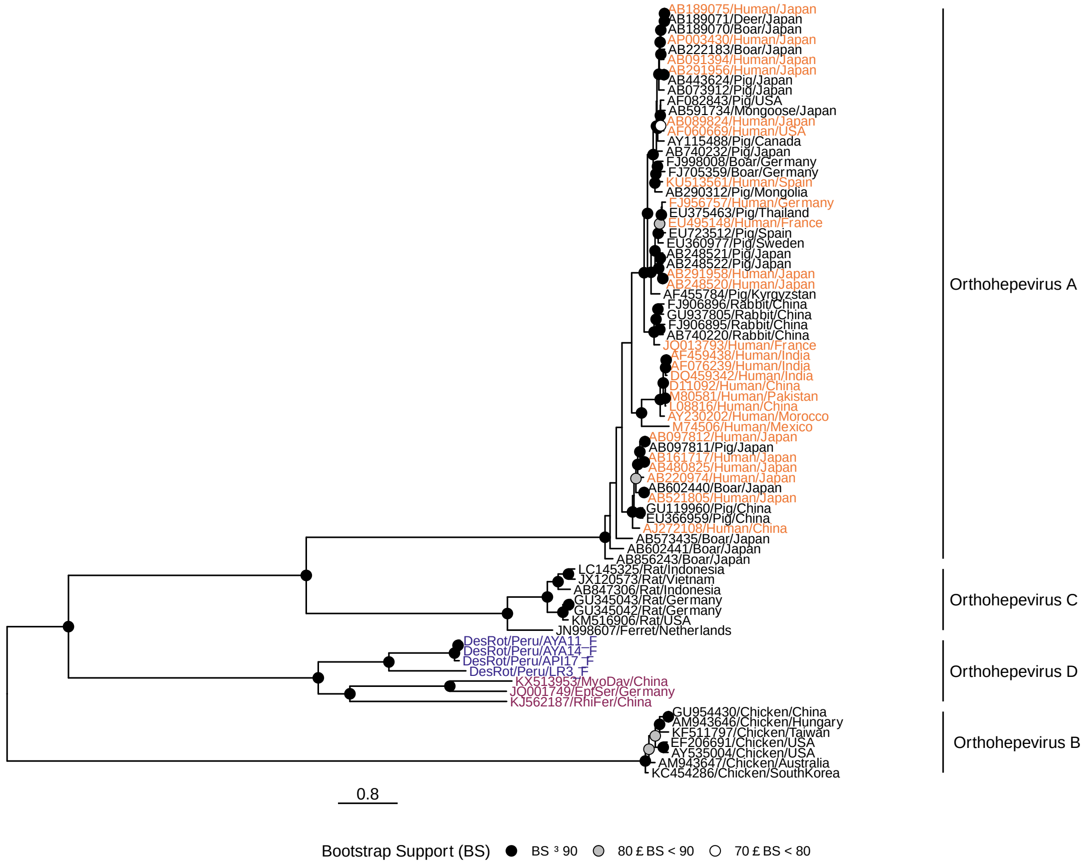

Figure S2. ***Hepeviridae* full genome phylogeny**. The maximum likelihood phylogeny was based on a 6,038 nucleotide alignment of 76 complete genome sequences. Phylogenetic analysis was performed in RAxML using the GTR+I+G substitution model and 100 bootstrap replicates. Sequences are colored to indicate vampire bat-associated HEV sequences (indigo), other bat-associated sequences (purple), human-associated sequences (orange) and sequences from other hosts (black). ICTV recognized species are shown along the right of the tree. The scale bar represents the mean number of substitutions per site.

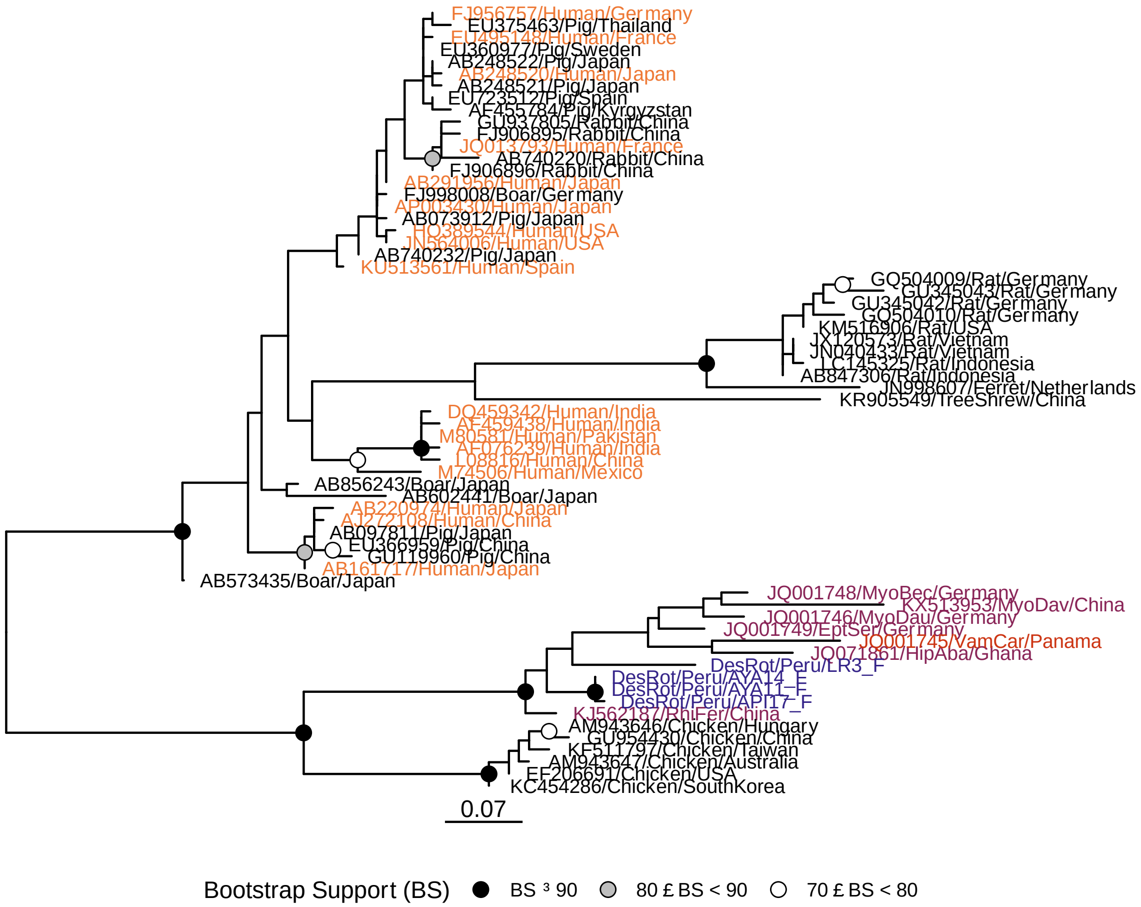

Figure S3. ***Hepeviridae* RdRp phylogeny.** The maximum likelihood phylogeny was based on a 109 amino acid alignment of 65 RdRp sequences (ORF1-1419-ORF1-1527). Phylogenetic analysis was performed in RAxML using the LG+G substitution model and 100 bootstrap replicates. Sequences are colored to indicate vampire bat-associated HEV sequences (indigo), sequences associated with other Neotropical bats (red), non-Neotropical bat-associated sequences (purple), human-associated sequences (orange) and sequences from other hosts (black). The scale bar represents the mean number of substitutions per site.

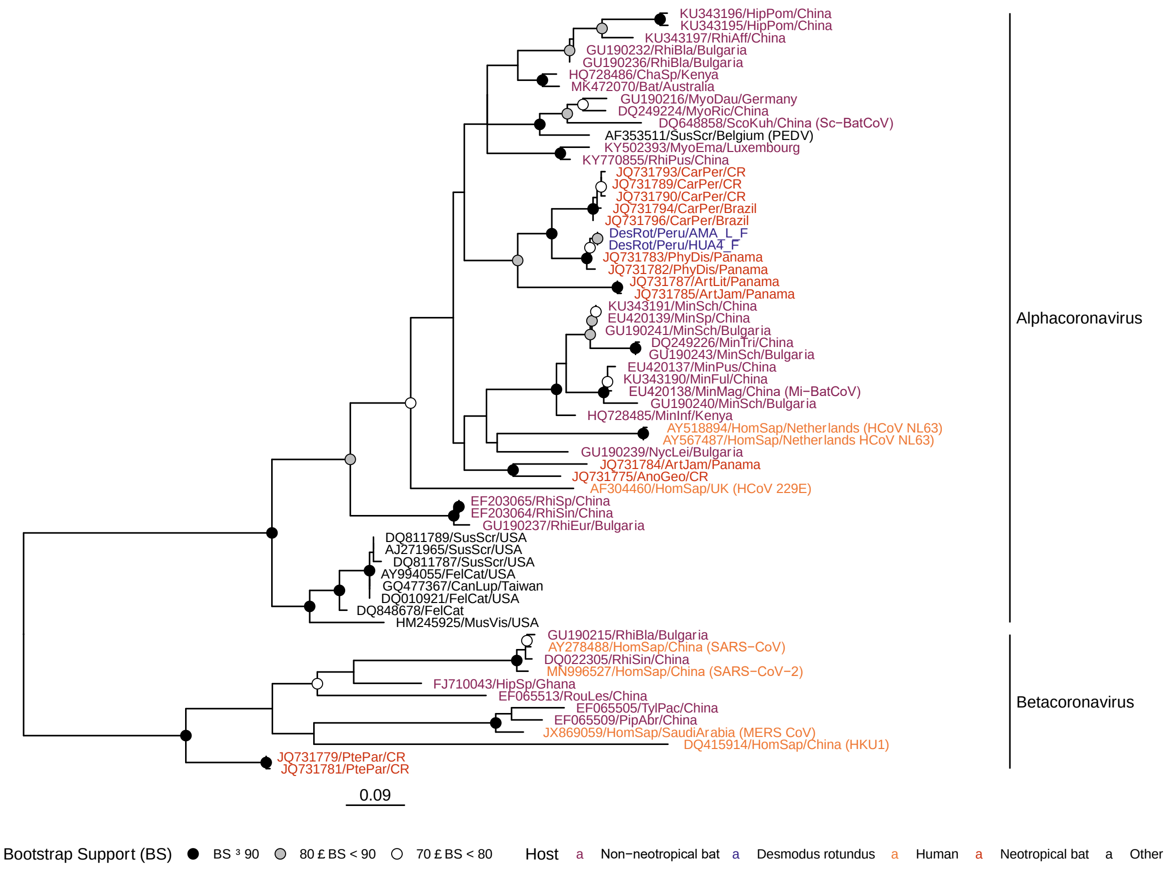

Figure S4. ***Coronaviridae*** **RdRp phylogeny.** Maximum likelihood tree based on a 272 amino acid alignment of 63 RdRp sequences including vampire bat-associated CoV sequences (indigo), Neotropical bat-associated sequences (red), non-Neotropical bat-associated sequences (purple), human-associated sequences (orange) and sequences from other hosts (black). ICTV recognized CoV species are shown in parentheses following each branch tip name, and genera are shown on the right of the tree. Phylogenetic analysis was performed in RAxML using the LG+I+G substitution model and 1000 bootstrap replicates. The scale bar represents the mean number of substitutions per site.

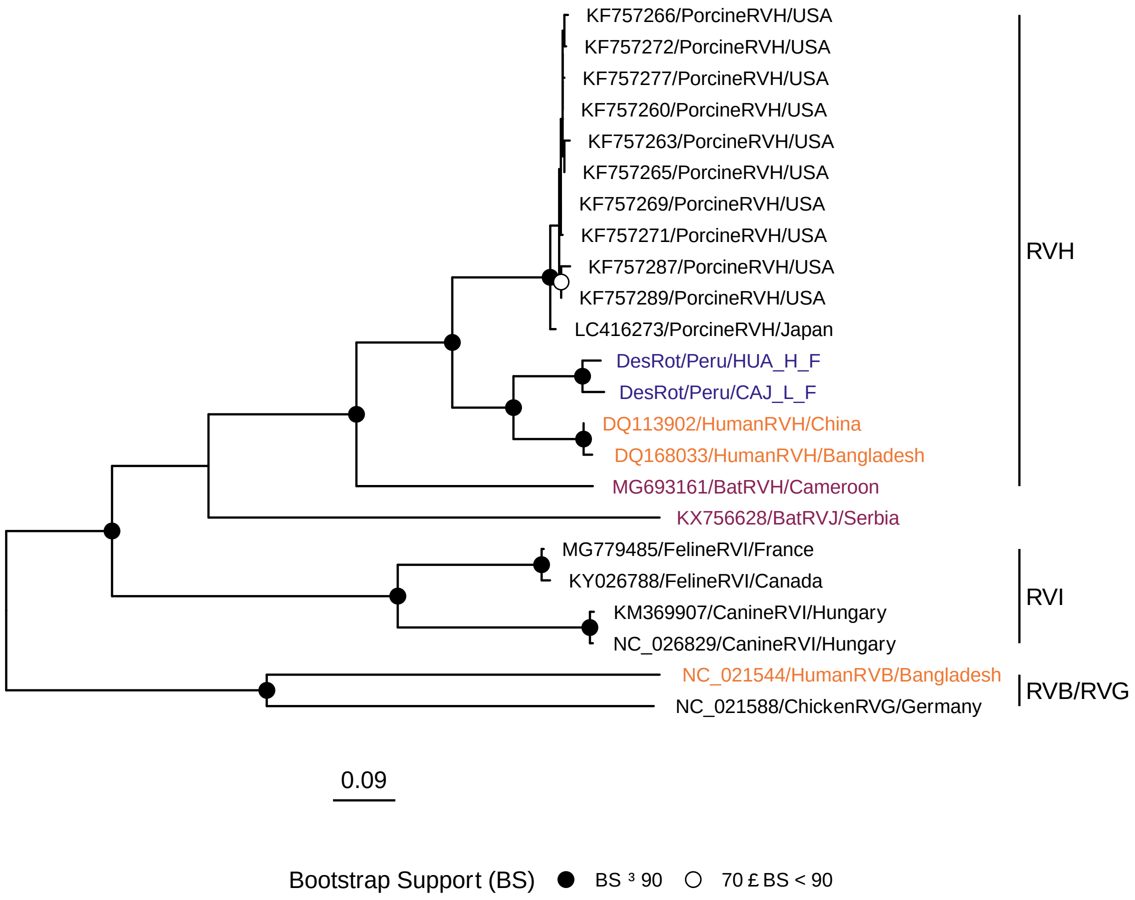

Figure S5. *Reoviridae* VP6 phylogeny. Maximum likelihood tree based on a 398 amino acid alignment of 22 sequences of the VP6 gene. Phylogenetic analysis was performed in RAxML using the LG+G+F substitution model and 100 bootstrap replicates. Analyses included two vampire bat-associated RVH sequences (indigo), other bat-associated RV sequences (purple), human-associated sequences (orange), and RV sequences from non-bat hosts (black). Although some antigenic types shown in the tree (RVG, RVH, RVI) are not yet formally recognized by the ICTV, they are shown on the right of the tree for context. The scale bar represents the mean number of substitutions per site.

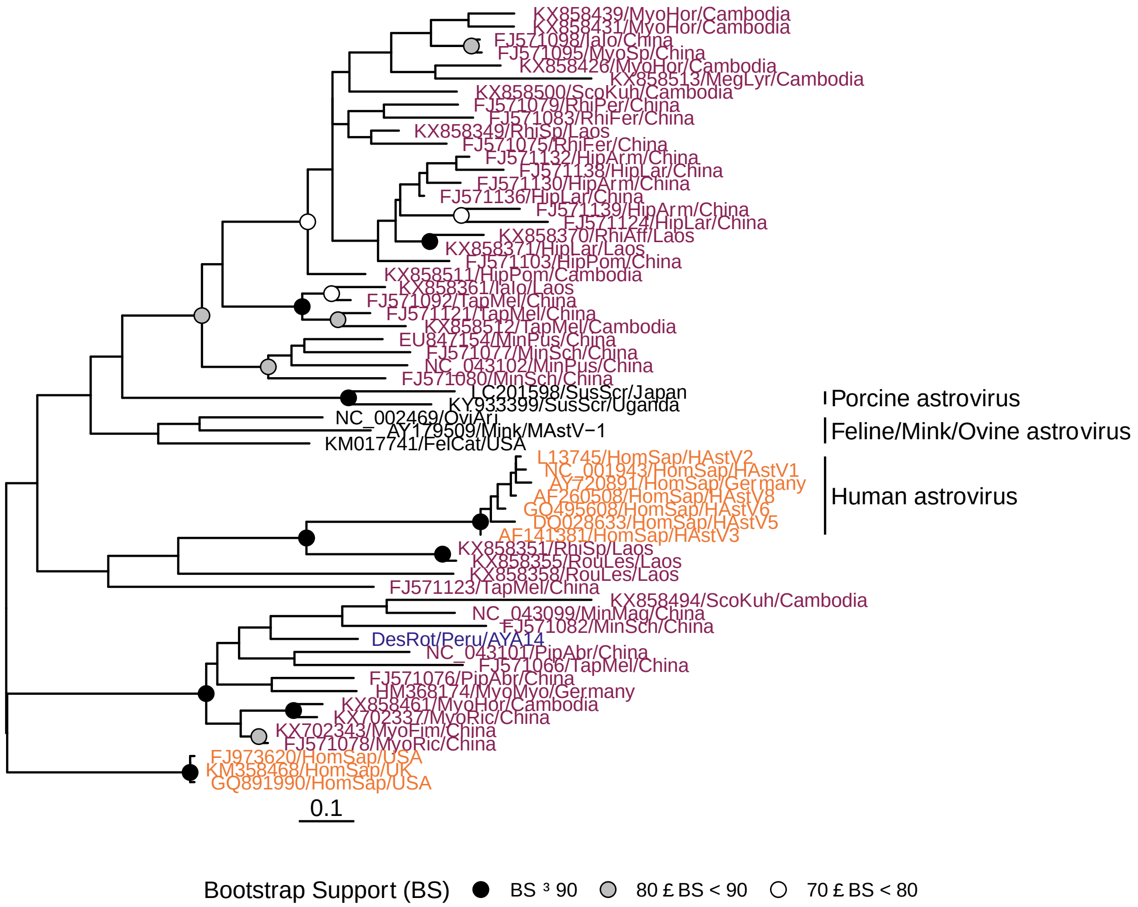

Figure S6. ***Astroviridae* phylogeny**. Maximum likelihood phylogeny based on a 128 amino acid alignment of 60 sequences of the RdRp. Phylogenetic analysis was performed in RAxML using the LG+I+G substitution model and 1000 bootstrap replicates. Analyses included one vampire bat-associated AstV sequence (indigo), other bat-associated AstV sequences (purple), human associated sequences (orange) and AstV sequences from non-bat hosts (black). ICTV recognized AstV species are shown on the right. The scale bar represents the mean number of substitutions per site

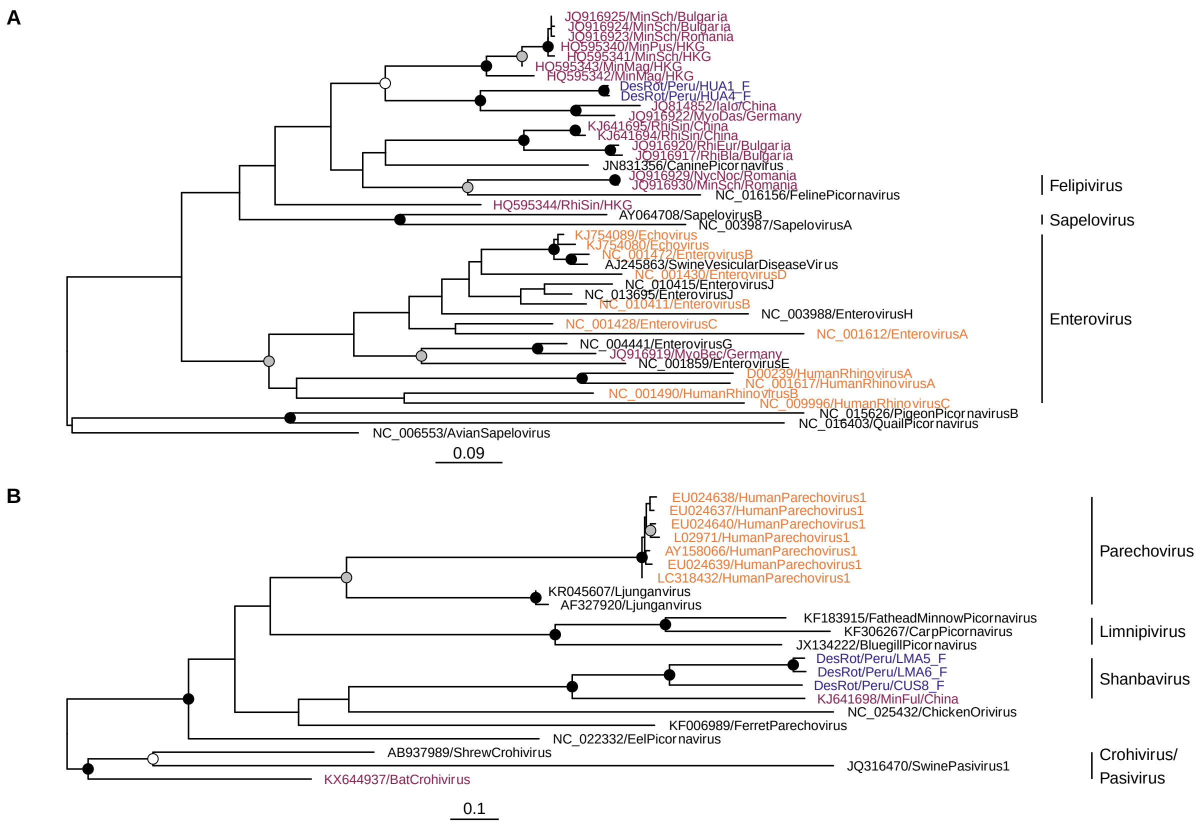

Figure S7. ***Picornaviridae* phylogenies**. Maximum likelihood trees of (**A**) *Enterovirus*-like viruses based on a 265 amino acid alignment of 43 sequences of the 3D polyprotein and (**B**) *Parechovirus*-like viruses based on a 396 amino acid alignment of 22 sequences of the 3D polyprotein. Phylogenetic analyses were performed in RAxML using the LG+I+G substitution model and 100 bootstrap replicates for both analyses. Colors indicate vampire bat-associated PicoV sequences (indigo), other bat-associated PicoV sequences (purple), human-associated sequences (orange) and PicoV sequences from non-bat hosts (black). ICTV recognized PicoV genera are shown on the right. The scale bars represent the mean number of substitutions per site.

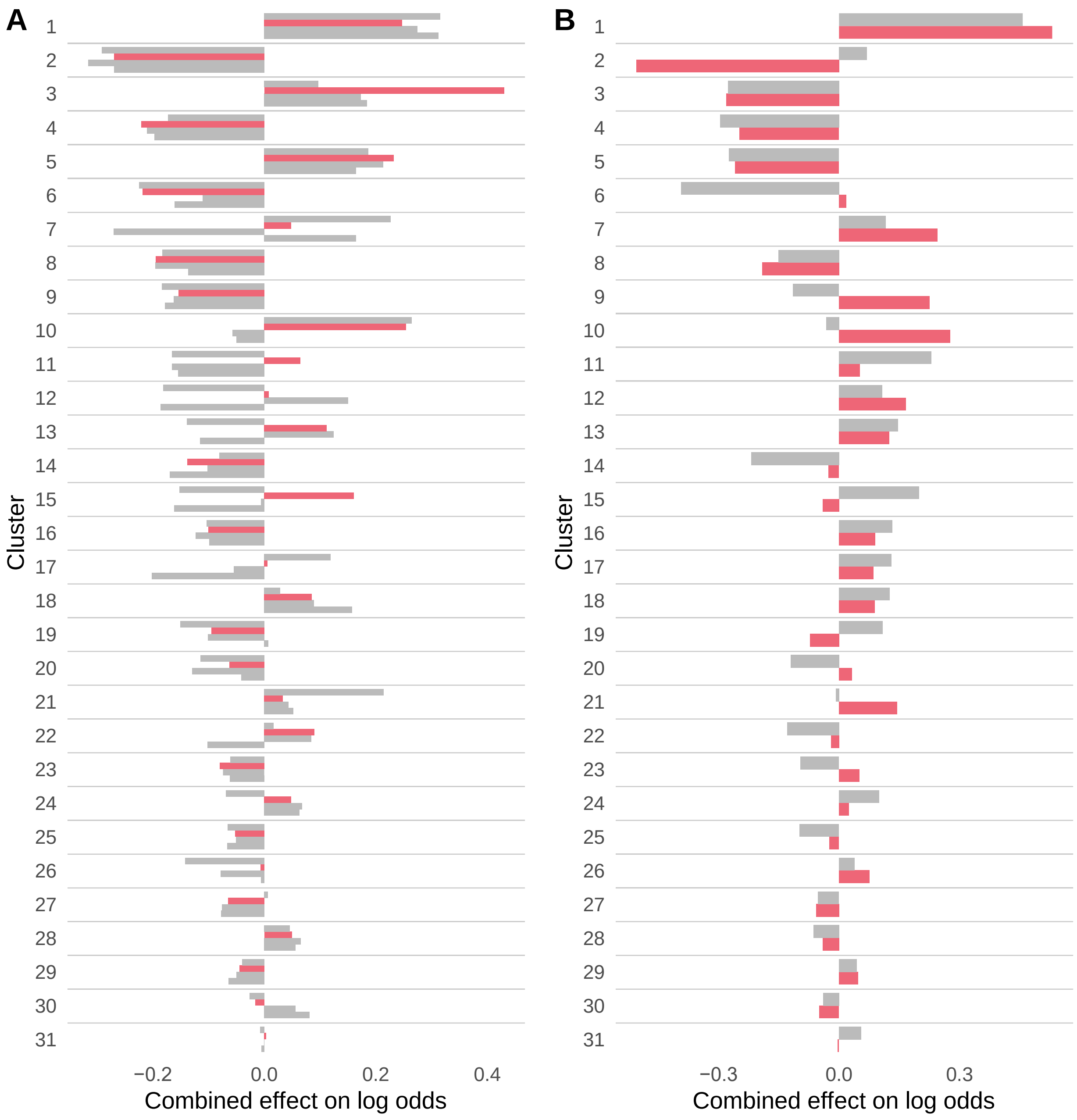

Figure S8. Effect of discrete clusters of correlated features on predicted scores for (A) *Hepeviridae* and (B) *Parechovirus*-like *Picornaviridae*. In each panel, the highest-ranked virus from that group is highlighted in red (AYA14_F_HEV and CUS8_F_PicoV in panel A and B, respectively). Clusters were taken from Mollentze et al. [15]. The influence of each cluster on the predictions of each virus was calculated by summing effect sizes across all features in the cluster.

Supplementary Tables

Table S1. Metagenomic datasets from which novel vampire bat viruses were characterized.

ENA Project ID	Accession	Pool alias	ENA Project ID	Accession	Pool alias
PRJEB28138	ERS2678722	AAC_H_F	PRJEB34487	ERS3786257	AMA2_F
	ERS2678723	AAC_H_SV		ERS3786258	AMA2_SV
	ERS2678724	AAC_L_F		ERS3786259	API140_F
	ERS2678725	AAC_L_SV		ERS3786260	API140_SV
	ERS2678727	AMA_L_F		ERS3786261	API141_F
	ERS2678728	AMA_L_SV		ERS3786262	API141_SV
	ERS2678729	CAJ_H_F		ERS3786263	API17_F
	ERS2678731	CAJ_H_SV		ERS3786264	API17_SV
	ERS2678733	CAJ_L_F		ERS3786265	API1_F
	ERS2678734	CAJ_L_SV		ERS3786266	API1_SV
	ERS2678735	HUA_H_F		ERS3786267	AYA11_F
	ERS2678736	HUA_H_SV		ERS3786268	AYA11_SV
	ERS2678738	LMA_L_F		ERS3786269	AYA12_F
	ERS2678740	LMA_L_SV		ERS3786270	AYA12_SV
	ERS2678742	LR_L_F		ERS3786271	AYA14_F
	ERS2678743	LR_L_SV		ERS3786272	AYA14_SV
				ERS3786273	AYA15_F
				ERS3786274	AYA15_SV
				ERS3786275	AYA1_F
				ERS3786276	AYA1_SV
				ERS3786277	AYA7_F
				ERS3786278	AYA7_SV
				ERS3786279	CAJ1_F
				ERS3786280	CAJ1_SV
				ERS3786281	CAJ2_F
				ERS3786282	CAJ2_SV
				ERS3786283	CAJ4_F
				ERS3786284	CAJ4_SV
				ERS3786285	CUS8_F
				ERS3786286	CUS8_SV
				ERS3786287	HUA1_F
				ERS3786288	HUA1_SV
				ERS3786289	HUA2_F
				ERS3786290	HUA2_SV
				ERS3786291	HUA3_F
				ERS3786292	HUA3_SV

		ERS3786293	HUA4_F
		ERS3786294	HUA4_SV
		ERS3786295	LMA5_F
		ERS3786296	LMA5_SV
		ERS3786297	LMA6_F
		ERS3786298	LMA6_SV
		ERS3786299	LR2_F
		ERS3786300	LR2_SV
		ERS3786301	LR3_F
		ERS3786302	LR3_SV

Table S2. Viral sequences examined in this study, sampling details, and associated Genbank and ENA accessions.

Virus	Sequence	Pool	Sample type	Colony/Colonies	Reads - ENA accession	Virus Genbank accession
Rabies virus	Nucleoprotein	CAJ4_SV	Saliva	CAJ4	ERS3786284	MW249021
	Nucleoprotein	HUA1_SV	Saliva	HUA1	ERS3786288	MW249020
	Partial nucleoprotein	HUA1_F	Feces	HUA1	ERS3786287	MW249019
Hepeviridae	Genome	AYA11_F	Feces	AYA11	ERS3786267	MW249012
	Genome	AYA14_F	Feces	AYA14	ERS3786271	MW249013
	Genome	API17_F	Feces	API17	ERS3786263	MW249011
	Genome	LR3_F	Feces	LR3	ERS3786301	MW249014
Coronaviridae	Genome	AMA_L_ F	Feces	AMA2, AMA6	ERS2678727	MT663548
	Partial genome	HUA4_F	Feces	HUA4	ERS3786293	MW249018
Reoviridae	VP1	CAJ_L_F	Feces	CAJ4	ERS2678733	MW249027
	VP2	CAJ_L_F	Feces	CAJ4	ERS2678733	MW249028
	VP3	CAJ_L_F	Feces	CAJ4	ERS2678733	MW249029
	VP4	CAJ_L_F	Feces	CAJ4	ERS2678733	MW259060
	VP6	CAJ_L_F	Feces	CAJ4	ERS2678733	MW249030
	NSP1	CAJ_L_F	Feces	CAJ4	ERS2678733	MW249022
	NSP2	CAJ_L_F	Feces	CAJ4	ERS2678733	MW249023
	NSP3	CAJ_L_F	Feces	CAJ4	ERS2678733	MW249024
	NSP4	CAJ_L_F	Feces	CAJ4	ERS2678733	MW249025
	NSP5	CAJ_L_F	Feces	CAJ4	ERS2678733	MW249026
	VP1	HUA_H_F	Feces	HUA1, HUA2	ERS2678735	MW249036
	VP2	HUA_H_F	Feces	HUA1, HUA2	ERS2678735	MW249037
	VP3	HUA_H_F	Feces	HUA1, HUA2	ERS2678735	MW249038
	VP4	HUA_H_F	Feces	HUA1, HUA2	ERS2678735	MW249039
	VP6	HUA_H_F	Feces	HUA1, HUA2	ERS2678735	MW249040
	NSP1	HUA_H_F	Feces	HUA1, HUA2	ERS2678735	MW249031
	NSP2	HUA_H_F	Feces	HUA1, HUA2	ERS2678735	MW249032
	NSP3	HUA_H_F	Feces	HUA1, HUA2	ERS2678735	MW249033
	NSP4	HUA_H_F	Feces	HUA1, HUA2	ERS2678735	MW249034
	NSP5	HUA_H_F	Feces	HUA1, HUA2	ERS2678735	MW249035
Picornaviridae	Partial genome	HUA1_F	Feces	HUA1	ERS3786287	MW249016
	Partial genome	HUA4_F	Feces	HUA4	ERS3786293	MW259061
	Partial genome	AYA12_F	Feces	AYA12	ERS3786269	MW259062
	Partial genome	API141_F	Feces	API141	ERS3786261	MW259063
	Partial genome	LMA5_F	Feces	LMA5	ERS3786295	MW259064
	Partial genome	LMA6_F	Feces	LMA6	ERS3786297	MW249015
	Partial genome	CUS8_F	Feces	CUS8	ERS3786285	MW249017
Astroviridae	Genome	AYA14_F	Feces	AYA14	ERS3786271	MW249010

Table S3. Genbank accessions, viral family and host for viral taxa included in phylogenies.

Virus ID	Viral family	Host	Accession
KJ562187/RhiFer/China	*Hepeviridae*	*Rhinolophus ferrumequinum*	KJ562187
JQ001748/MyoBec/Germany	*Hepeviridae*	*Myotis bechsteinii*	JQ001748
JQ001746/MyoDau/Germany	*Hepeviridae*	*Myotis daubentonii*	JQ001746
JQ001749/EptSer/Germany	*Hepeviridae*	*Eptesicus serotinus*	JQ001749
KX513953/MyoDav/China	*Hepeviridae*	*Myotis davidii*	KX513953
JQ071861/HipAba/Ghana	*Hepeviridae*	*Hipposideros abae*	JQ071861
JQ001745/VamCar/Panama	*Hepeviridae*	*Vampyrodes caraccioli*	JQ001745
EF206691/Chicken/USA	*Hepeviridae*	Chicken	EF206691
KC454286/Chicken/SouthKorea	*Hepeviridae*	Chicken	KC454286
AM943647/Chicken/Australia	*Hepeviridae*	Chicken	AM943647
KF511797/Chicken/Taiwan	*Hepeviridae*	Chicken	KF511797
AM943646/Chicken/Hungary	*Hepeviridae*	Chicken	AM943646
GU954430/Chicken/China	*Hepeviridae*	Chicken	GU954430
AP003430/Human/Japan	*Hepeviridae*	Human	AP003430
JN564006/Human/USA	*Hepeviridae*	Human	JN564006
AB740232/Pig/Japan	*Hepeviridae*	Pig	AB740232
HQ389544/Human/USA	*Hepeviridae*	Human	HQ389544
FJ998008/Boar/Germany	*Hepeviridae*	Boar	FJ998008
AB291956/Human/Japan	*Hepeviridae*	Human	AB291956
AB073912/Pig/Japan	*Hepeviridae*	Pig	AB073912
KU513561/Human/Spain	*Hepeviridae*	Human	KU513561
AB248520/Human/Japan	*Hepeviridae*	Human	AB248520
AB248521/Pig/Japan	*Hepeviridae*	Pig	AB248521
AB248522/Pig/Japan	*Hepeviridae*	Pig	AB248522
AF455784/Pig/Kyrgyzstan	*Hepeviridae*	Pig	AF455784
EU723512/Pig/Spain	*Hepeviridae*	Pig	EU723512
FJ956757/Human/Germany	*Hepeviridae*	Human	FJ956757
EU360977/Pig/Sweden	*Hepeviridae*	Pig	EU360977
EU495148/Human/France	*Hepeviridae*	Human	EU495148
EU375463/Pig/Thailand	*Hepeviridae*	Pig	EU375463
FJ906895/Rabbit/China	*Hepeviridae*	Rabbit	FJ906895
FJ906896/Rabbit/China	*Hepeviridae*	Rabbit	FJ906896
JQ013793/Human/France	*Hepeviridae*	Human	JQ013793
GU937805/Rabbit/China	*Hepeviridae*	Rabbit	GU937805
AB740220/Rabbit/China	*Hepeviridae*	Rabbit	AB740220
AB573435/Boar/Japan	*Hepeviridae*	Boar	AB573435
AB856243/Boar/Japan	*Hepeviridae*	Boar	AB856243
AB161717/Human/Japan	*Hepeviridae*	Human	AB161717

AB097811/Pig/Japan	*Hepeviridae*	Pig	AB097811
AJ272108/Human/China	*Hepeviridae*	Human	AJ272108
EU366959/Pig/China	*Hepeviridae*	Pig	EU366959
GU119960/Pig/China	*Hepeviridae*	Pig	GU119960
AB220974/Human/Japan	*Hepeviridae*	Human	AB220974
AB602441/Boar/Japan	*Hepeviridae*	Boar	AB602441
M80581/Human/Pakistan	*Hepeviridae*	Human	M80581
DQ459342/Human/India	*Hepeviridae*	Human	DQ459342
L08816/Human/China	*Hepeviridae*	Human	L08816
AF459438/Human/India	*Hepeviridae*	Human	AF459438
AF076239/Human/India	*Hepeviridae*	Human	AF076239
M74506/Human/Mexico	*Hepeviridae*	Human	M74506
GQ504009/Rat/Germany	*Hepeviridae*	Rat	GQ504009
JX120573/Rat/Vietnam	*Hepeviridae*	Rat	JX120573
JN040433/Rat/Vietnam	*Hepeviridae*	Rat	JN040433
LC145325/Rat/Indonesia	*Hepeviridae*	Rat	LC145325
AB847306/Rat/Indonesia	*Hepeviridae*	Rat	AB847306
KM516906/Rat/USA	*Hepeviridae*	Rat	KM516906
GU345042/Rat/Germany	*Hepeviridae*	Rat	GU345042
GQ504010/Rat/Germany	*Hepeviridae*	Rat	GQ504010
GU345043/Rat/Germany	*Hepeviridae*	Rat	GU345043
JN998607/Ferret/Netherlands	*Hepeviridae*	Ferret	JN998607
KR905549/TreeShrew/China	*Hepeviridae*	Tree Shrew	KR905549
AB091394/Human/Japan	*Hepeviridae*	Human	AB091394
AB222183/Boar/Japan	*Hepeviridae*	Boar	AB222183
AB189070/Boar/Japan	*Hepeviridae*	Boar	AB189070
AB189071/Deer/Japan	*Hepeviridae*	Deer	AB189071
AB189075/Human/Japan	*Hepeviridae*	Human	AB189075
AB443624/Pig/Japan	*Hepeviridae*	Pig	AB443624
AB089824/Human/Japan	*Hepeviridae*	Human	AB089824
AF082843/Pig/USA	*Hepeviridae*	Pig	AF082843
AF060669/Human/USA	*Hepeviridae*	Human	AF060669
AB591734/Mongoose/Japan	*Hepeviridae*	Mongoose	AB591734
AY115488/Pig/Canada	*Hepeviridae*	Pig	AY115488
AB290312/Pig/Mongolia	*Hepeviridae*	Pig	AB290312
FJ705359/Boar/Germany	*Hepeviridae*	Boar	FJ705359
AB291958/Human/Japan	*Hepeviridae*	Human	AB291958
AB097812/Human/Japan	*Hepeviridae*	Human	AB097812
AB480825/Human/Japan	*Hepeviridae*	Human	AB480825
AB521805/Human/Japan	*Hepeviridae*	Human	AB521805

AB602440/Boar/Japan	*Hepeviridae*	Boar	AB602440
D11092/Human/China	*Hepeviridae*	Human	D11092
AY230202/Human/Morocco	*Hepeviridae*	Human	AY230202
AY535004/Chicken/USA	*Hepeviridae*	Chicken	AY535004
JQ731783/PhyDis/Panama	*Coronaviridae*	*Phyllostomus discolor*	JQ731783
JQ731782/PhyDis/Panama	*Coronaviridae*	*Phyllostomus discolor*	JQ731782
JQ731789/CarPer/CR	*Coronaviridae*	*Carollia perspicillata*	JQ731789
JQ731790/CarPer/CR	*Coronaviridae*	*Carollia perspicillata*	JQ731790
JQ731793/CarPer/CR	*Coronaviridae*	*Carollia perspicillata*	JQ731793
JQ731796/CarPer/Brazil	*Coronaviridae*	*Carollia perspicillata*	JQ731796
JQ731794/CarPer/Brazil	*Coronaviridae*	*Carollia perspicillata*	JQ731794
JQ731785/ArtJam/Panama	*Coronaviridae*	*Artibeus jamaicensis*	JQ731785
JQ731787/ArtLit/Panama	*Coronaviridae*	*Artibeus lituratus*	JQ731787
KY502393/MyoEma/Luxembourg	*Coronaviridae*	*Myotis emarginatus*	KY502393
KY770855/RhiPus/China	*Coronaviridae*	*Rhinolophus pusillus*	KY770855
GU190236/RhiBla/Bulgaria	*Coronaviridae*	*Rhinolophus blasii*	GU190236
GU190232/RhiBla/Bulgaria	*Coronaviridae*	*Rhinolophus blasii*	GU190232
KU343197/RhiAff/China	*Coronaviridae*	*Rhinolophus affinis*	KU343197
KU343196/HipPom/China	*Coronaviridae*	*Hipposideros pomona*	KU343196
KU343195/HipPom/China	*Coronaviridae*	*Hipposideros pomona*	KU343195
HQ728486/ChaSp/Kenya	*Coronaviridae*	*Chaerephon sp*	HQ728486
KU343190/MinFul/China	*Coronaviridae*	*Miniopterus fuliginosus*	KU343190
EU420138/MinMag/China	*Coronaviridae*	*Miniopterus magnater*	EU420138
GU190240/MinSch/Bulgaria	*Coronaviridae*	*Miniopterus schreibersii*	GU190240
GU190243/MinSch/Bulgaria	*Coronaviridae*	*Miniopterus schreibersii*	GU190243
DQ249226/MinTri/China	*Coronaviridae*	*Miniopterus magnater*	DQ249226
KU343191/MinSch/China	*Coronaviridae*	*Miniopterus schreibersii*	KU343191
GU190241/MinSch/Bulgaria	*Coronaviridae*	*Miniopterus schreibersii*	GU190241
HQ728485/MinInf/Kenya	*Coronaviridae*	*Miniopterus inflatus*	HQ728485
GU190239/NycLei/Bulgaria	*Coronaviridae*	*Nyctalus leisleri*	GU190239
DQ249224/MyoRic/China	*Coronaviridae*	*Myotis ricketti*	DQ249224
GU190216/MyoDau/Germany	*Coronaviridae*	*Myotis daubentonii*	GU190216
DQ648858/ScoKuh/China	*Coronaviridae*	*Scotophilus kuhlii*	DQ648858
AF353511/SusScr/Belgium	*Coronaviridae*	Pig	AF353511
JQ731775/AnoGeo/CR	*Coronaviridae*	*Anoura geoffroyi*	JQ731775
JQ731784/ArtJam/Panama	*Coronaviridae*	*Artibeus jamaicensis*	JQ731784
AY567487/HomSap/Netherlands	*Coronaviridae*	Human	AY567487
AY518894/HomSap/Netherlands	*Coronaviridae*	Human	AY518894
HM245925/MusVis/USA	*Coronaviridae*	American Mink	HM245925
DQ010921/FelCat/USA	*Coronaviridae*	Cat	DQ010921

DQ811789/SusScr/USA	*Coronaviridae*	Pig	DQ811789
GU190237/RhiEur/Bulgaria	*Coronaviridae*	*Rhinolophus euryale*	GU190237
EF203064/RhiSin/China	*Coronaviridae*	*Rhinolophus sinicus*	EF203064
AF304460/HomSap/UK	*Coronaviridae*	Human	AF304460
EF065505/TylPac/China	*Coronaviridae*	*Tylonycteris pachypus*	EF065505
EF065509/PipAbr/China	*Coronaviridae*	*Pipistrellus abramus*	EF065509
JX869059/HomSap/SaudiArabia	*Coronaviridae*	Human	JX869059
GU190215/RhiBla/Bulgaria	*Coronaviridae*	*Rhinolophus blasii*	GU190215
DQ022305/RhiSin/China	*Coronaviridae*	*Rhinolophus sinicus*	DQ022305
FJ710043/HipSp/Ghana	*Coronaviridae*	*Hipposideros sp*	FJ710043
EF065513/RouLes/China	*Coronaviridae*	*Rousettus lechenaulti*	EF065513
JQ731781/PtePar/CR	*Coronaviridae*	*Pteronotus parnellii*	JQ731781
JQ731779/PtePar/CR	*Coronaviridae*	*Pteronotus parnellii*	JQ731779
DQ415914/HomSap/China	*Coronaviridae*	Human	DQ415914
KX756628/BatRVJ/Serbia	*Reoviridae*	*Miniopterus schreibersii*	KX756628
MG693161/BatRVH/Cameroon	*Reoviridae*	*Eidolon helvum*	MG693161
DQ113902/HumanRVH/China	*Reoviridae*	Human	DQ113902
DQ168033/HumanRVH/Bangladesh	*Reoviridae*	Human	DQ168033
KF757260/PorcineRVH/USA	*Reoviridae*	Pig	KF757260
KF757265/PorcineRVH/USA	*Reoviridae*	Pig	KF757265
KF757277/PorcineRVH/USA	*Reoviridae*	Pig	KF757277
KF757269/PorcineRVH/USA	*Reoviridae*	Pig	KF757269
KF757271/PorcineRVH/USA	*Reoviridae*	Pig	KF757271
KF757272/PorcineRVH/USA	*Reoviridae*	Pig	KF757272
KF757289/PorcineRVH/USA	*Reoviridae*	Pig	KF757289
KF757263/PorcineRVH/USA	*Reoviridae*	Pig	KF757263
KF757266/PorcineRVH/USA	*Reoviridae*	Pig	KF757266
LC416273/PorcineRVH/Japan	*Reoviridae*	Pig	LC416273
KF757287/PorcineRVH/USA	*Reoviridae*	Pig	KF757287
NC_026829/CanineRVI/Hungary	*Reoviridae*	Dog	NC_026829
KM369907/CanineRVI/Hungary	*Reoviridae*	Dog	KM369907
KY026788/FelineRVI/Canada	*Reoviridae*	Cat	KY026788
MG779485/FelineRVI/France	*Reoviridae*	Cat	MG779485
NC_021544/HumanRVB/Bangladesh	*Reoviridae*	Human	NC_021544
NC_021588/ChickenRVG/Germany	*Reoviridae*	Chicken	NC_021588
HQ595344/RhiSin/HKG	*Picornaviridae*	*Rhinolophus sinicus*	HQ595344
JQ814852/IaIo/China	*Picornaviridae*	*Ia io*	JQ814852
JQ916922/MyoDas/Germany	*Picornaviridae*	*Myotis dasycneme*	JQ916922
KJ641694/RhiSin/China	*Picornaviridae*	*Rhinolophus sinicus*	KJ641694
KJ641695/RhiSin/China	*Picornaviridae*	*Rhinolophus sinicus*	KJ641695

JQ916917/RhiBla/Bulgaria	*Picornaviridae*	*Rhinolophus blasii*	JQ916917
JQ916920/RhiEur/Bulgaria	*Picornaviridae*	*Rhinolophus euryale*	JQ916920
JQ916924/MinSch/Bulgaria	*Picornaviridae*	*Miniopterus schreibersii*	JQ916924
JQ916925/MinSch/Bulgaria	*Picornaviridae*	*Miniopterus schreibersii*	JQ916925
JQ916923/MinSch/Romania	*Picornaviridae*	*Miniopterus schreibersii*	JQ916923
HQ595341/MinSch/HKG	*Picornaviridae*	*Miniopterus schreibersii*	HQ595341
HQ595340/MinPus/HKG	*Picornaviridae*	*Miniopterus pusillus*	HQ595340
HQ595343/MinMag/HKG	*Picornaviridae*	*Miniopterus magnater*	HQ595343
HQ595342/MinMag/HKG	*Picornaviridae*	*Miniopterus magnater*	HQ595342
JN831356/CaninePicornavirus	*Picornaviridae*	Dog	JN831356
JQ916930/MinSch/Romania	*Picornaviridae*	*Miniopterus schreibersii*	JQ916930
JQ916929/NycNoc/Romania	*Picornaviridae*	*Nyctalus noctula*	JQ916929
NC_016156/FelinePicornavirus	*Picornaviridae*	Cat	NC_016156
NC_001612/EnterovirusA	*Picornaviridae*	Human	NC_001612
NC_001490/HumanRhinovirusB	*Picornaviridae*	Human	NC_001490
NC_003988/EnterovirusH	*Picornaviridae*	Simian	NC_003988
KJ754089/Echovirus	*Picornaviridae*	Human	KJ754089
KJ754080/Echovirus	*Picornaviridae*	Human	KJ754080
NC_001472/EnterovirusB	*Picornaviridae*	Human	NC_001472
AJ245863/SwineVesicularDiseaseVirus	*Picornaviridae*	Porcine	AJ245863
NC_010415/EnterovirusJ	*Picornaviridae*	Simian	NC_010415
NC_013695/EnterovirusJ	*Picornaviridae*	Simian	NC_013695
NC_010411/EnterovirusB	*Picornaviridae*	Human	NC_010411
NC_001430/EnterovirusD	*Picornaviridae*	Human	NC_001430
NC_001428/EnterovirusC	*Picornaviridae*	Human	NC_001428
NC_004441/EnterovirusG	*Picornaviridae*	Porcine	NC_004441
JQ916919/MyoBec/Germany	*Picornaviridae*	*Myotis bechsteinii*	JQ916919
NC_001859/EnterovirusE	*Picornaviridae*	Bovine	NC_001859
NC_009996/HumanRhinovirusC	*Picornaviridae*	Human	NC_009996
NC_003987/SapelovirusA	*Picornaviridae*	Porcine	NC_003987
AY064708/SapelovirusB	*Picornaviridae*	Simian	AY064708
NC_006553/AvianSapelovirus	*Picornaviridae*	Duck	NC_006553
NC_001617/HumanRhinovirusA	*Picornaviridae*	Human	NC_001617
D00239/HumanRhinovirusA	*Picornaviridae*	Human	D00239
NC_015626/PigeonPicornavirusB	*Picornaviridae*	Pigeon	NC_015626
NC_016403/QuailPicornavirus	*Picornaviridae*	Quail	NC_016403
KJ641698/MinFul	*Picornaviridae*	*Miniopterus fuliginosus*	KJ641698
NC_022332/EelPicornavirus	*Picornaviridae*	Eel	NC_022332
L02971/HumanParechovirus1	*Picornaviridae*	Human	L02971
AY158066/HumanParechovirus1	*Picornaviridae*	Human	AY158066

LC318432/HumanParechovirus1	*Picornaviridae*	Human	LC318432
EU024639/HumanParechovirus1	*Picornaviridae*	Human	EU024639
EU024640/HumanParechovirus1	*Picornaviridae*	Human	EU024640
EU024637/HumanParechovirus1	*Picornaviridae*	Human	EU024637
EU024638/HumanParechovirus1	*Picornaviridae*	Human	EU024638
AF327920/Ljunganvirus	*Picornaviridae*	Ljunganvirus	AF327920
KR045607/Ljunganvirus	*Picornaviridae*	Ljunganvirus	KR045607
KF006989/FerretParechovirus	*Picornaviridae*	Ferret	KF006989
AB937989/ShrewCrohivirus	*Picornaviridae*	Shrew	AB937989
KX644937/BatCrohivirus	*Picornaviridae*	Eidolon helvum	KX644937
JX134222/BluegillPicornavirus	*Picornaviridae*	Bluegill Fish	JX134222
KF183915/FatheadMinnowPicornavirus	*Picornaviridae*	Fathead Minnow	KF183915
KF306267/CarpPicornavirus	*Picornaviridae*	Carp	KF306267
NC_025432/ChickenOrivirus	*Picornaviridae*	Chicken	NC_025432
JQ316470/SwinePasivirus1	*Picornaviridae*	Pig	JQ316470
KX858431/MyoHor/Cambodia	*Astroviridae*	*Myotis horsfieldii*	KX858431
FJ571095/MyoSp/China	*Astroviridae*	*Myotis spp*	FJ571095
FJ571098/IaIo/China	*Astroviridae*	*Ia io*	FJ571098
KX858439/MyoHor/Cambodia	*Astroviridae*	*Myotis horsfieldii*	KX858439
KX858511/HipPom/Cambodia	*Astroviridae*	*Hipposideros pomona*	KX858511
FJ571130/HipArm/China	*Astroviridae*	*Hipposideros armiger*	FJ571130
FJ571136/HipLar/China	*Astroviridae*	*Hipposideros larvatus*	FJ571136
FJ571132/HipArm/China	*Astroviridae*	*Hipposideros armiger*	FJ571132
FJ571138/HipLar/China	*Astroviridae*	*Hipposideros larvatus*	FJ571138
KX858370/RhiAff/Laos	*Astroviridae*	*Rhinolophus affinis*	KX858370
KX858371/HipLar/Laos	*Astroviridae*	*Hipposideros larvatus*	KX858371
KX858349/RhiSp/Laos	*Astroviridae*	*Rhinolophus spp*	KX858349
FJ571075/RhiFer/China	*Astroviridae*	*Rhinolophus ferrumequinum*	FJ571075
FJ571103/HipPom/China	*Astroviridae*	*Hipposideros pomona*	FJ571103
FJ571079/RhiPer/China	*Astroviridae*	*Rhinolophus pearsonii*	FJ571079
KX858426/MyoHor/Cambodia	*Astroviridae*	*Myotis horsfieldii*	KX858426
FJ571139/HipArm/China	*Astroviridae*	*Hipposideros armiger*	FJ571139
FJ571124/HipLar/China	*Astroviridae*	*Hipposideros larvatus*	FJ571124
FJ571083/RhiFer/China	*Astroviridae*	*Rhinolophus ferrumequinum*	FJ571083
KX858500/ScoKuh/Cambodia	*Astroviridae*	*Scotophilus kuhlii*	KX858500
KX858513/MegLyr/Cambodia	*Astroviridae*	*Megaderma lyra*	KX858513
FJ571092/TapMel/China	*Astroviridae*	*Taphozous melanopogon*	FJ571092
KX858361/IaIo/Laos	*Astroviridae*	*Ia io*	KX858361
KX858512/TapMel/Cambodia	*Astroviridae*	*Taphozous melanopogon*	KX858512
FJ571121/TapMel/China	*Astroviridae*	*Taphozous melanopogon*	FJ571121

FJ571077/MinSch/China	*Astroviridae*	*Miniopterus schreibersi*	FJ571077
EU847154/MinPus/China	*Astroviridae*	*Miniopterus pusillus*	EU847154
NC_043102/MinPus/China	*Astroviridae*	*Miniopterus pusillus*	NC_043102
FJ571080/MinSch/China	*Astroviridae*	*Miniopterus schreibersi*	FJ571080
NC_002469/OviAri	*Astroviridae*	Sheep	NC_002469
KM017741/FelCat/USA	*Astroviridae*	Cat	KM017741
FJ973620/HomSap/USA	*Astroviridae*	Human	FJ973620
KM358468/HomSap/UK	*Astroviridae*	Human	KM358468
GQ891990/HomSap/USA	*Astroviridae*	Human	GQ891990
KY933399/SusScr/Uganda	*Astroviridae*	Pig	KY933399
LC201598/SusScr/Japan	*Astroviridae*	Pig	LC201598
FJ571123/TapMel/China	*Astroviridae*	*Taphozous melanopogon*	FJ571123
KX702337/MyoRic/China	*Astroviridae*	*Myotis ricketti*	KX702337
KX858461/MyoHor/Cambodia	*Astroviridae*	*Myotis horsfieldii*	KX858461
KX702343/MyoFim/China	*Astroviridae*	*Myotis fimbriatus*	KX702343
FJ571078/MyoRic/China	*Astroviridae*	*Myotis ricketti*	FJ571078
HM368174/MyoMyo/Germany	*Astroviridae*	*Myotis myotis*	HM368174
FJ571076/PipAbr/China	*Astroviridae*	*Pipistrellus abramus*	FJ571076
NC_043101/PipAbr/China	*Astroviridae*	*Pipistrellus abramus*	NC_043101
FJ571066/TapMel/China	*Astroviridae*	*Taphozous melanopogon*	FJ571066
NC_043099/MinMag/China	*Astroviridae*	*Miniopterus magnater*	NC_043099
KX858494/ScoKuh/Cambodia	*Astroviridae*	*Scotophilus kuhlii*	KX858494
FJ571082/MinSch/China	*Astroviridae*	*Miniopterus schreibersi*	FJ571082
KX858358/RouLes/Laos	*Astroviridae*	*Rousettus leschenaultii*	KX858358
AY720891/HomSap/Germany	*Astroviridae*	Human	AY720891
NC_001943/HomSap/UK	*Astroviridae*	Human	NC_001943
KX858355/RouLes/Laos	*Astroviridae*	*Rousettus leschenaultii*	KX858355
KX858351/RhiSp/Laos	*Astroviridae*	*Rhinolophus spp*	KX858351

Table S4. Pairwise identities for each segment between novel vampire bat RVH sequences, the closely related human RVH B219 and bat RVH which was not included in the phylogeny.

Segment	Segment lengths		Pairwise % ID Peru bat RVH	Pairwise % ID Human RVH B219			Pairwise % ID Korean bat RVH		
	HUA_H	CAJ_L		HUA_H	CAJ_L	Alignment length (bp)	HUA_H	CAJ_L	Alignment length (bp)
VP1	3507	3038	65.4	70.6	56.9	3538	63.4	62.3	268
VP2	2890	2440	65.2	71.5	59.5	3005	-	-	-
VP3	2219	1414	77.8	58	49.4	2304	66.2	58	340
VP4	2518	2349	42.1	57.2	32.8	2598	65.3	65.4	202
VP6	1252	1242	94.8	72.1	72.6	1289	-	-	-
NSP1	1386	1253	71.1	57.8	58.4	1389	-	-	-
NSP2	934	928	96.1	70.7	71.2	1016	-	-	-
NSP3	872	794	86.5	62	64.1	938	-	-	-
NSP4	735	598	87.7	40.9	45	829	-	-	-
NSP5	648	783	87.3	67.9	63.2	783	-	-	-

Table S5. Similarities between *Parechovirus*-like sequences in vampire bats from different locations.

	API141_F*	AYA12_F	LMA5_F	LMA6_F	CUS8_F
API141_F		95.8	74.2	74.8	65.5
AYA12_F	99.1		74.5	75.1	64.6
LMA5_F	82.9	83.8		98.5	60.7
LMA6_F	83.8	84.7	98.2		61.9
CUS8_F	72.1	72.1	72.1	73	

*Nucleotide identities of 333 bp alignment are shown above the diagonal and protein identities of 111 aa alignment are shown below.
